# Age-driven shifts of the camel gut microbiome and resistome in extensively reared dromedary camels

**DOI:** 10.1128/spectrum.03183-25

**Published:** 2026-06-03

**Authors:** Wenjing Wang, Yitao Li, Yaqian Liang, Junkai Wang, Zhihao Zhang, Yan Zhang, Chencheng Xiao, Haihong Hao

**Affiliations:** 1 College of Animal Science and Technology, Shihezi University, Xinjiang, China; 2 National Key Laboratory of Agricultural Microbiology Resources Exploration and Utilization, Huazhong Agricultural University, Hubei, China; Yangzhou University, Yangzhou, Jiangsu, China

**Keywords:** gut microbiota, antimicrobial resistance, metagenomics, age-dependent adaptation, mobile genetic elements

## Abstract

Camels are uniquely adapted to arid environments and are commonly raised in extensive grazing systems. The composition of their gut microbiome and antimicrobial resistance genes (ARGs) is expected to change with host development, but age-related patterns have not been well described. In this study, we analyzed fecal samples from juvenile (approximately 6 months old) and adult (6 years) dromedary camels kept under the same grazing management, with no recorded therapeutic antibiotic treatments during the study period. Shotgun metagenomic sequencing was used to profile bacterial communities, ARGs, and mobile genetic elements (MGEs). Juvenile camels showed lower alpha diversity and greater inter-individual variation than adults, and their gut communities were dominated by facultative anaerobes such as *Escherichia* and *Streptococcus*. Adult camels carried more stable, fiber-adapted communities enriched in Bacteroidaceae and Prevotellaceae. In parallel with these microbiome changes, the resistome also differed by age. Juveniles carried a wider range of ARGs, with higher contributions from multidrug efflux pumps and vancomycin resistance genes. Adults had a smaller and more concentrated set of ARGs, mainly β-lactamase and tetracycline resistance genes, together with lower ARG richness and diversity. MGEs also showed distinct age-related patterns: transposase genes were more common in juveniles, whereas insertion sequence-associated genes were more abundant in adults, suggesting age-specific routes of potential ARG mobility. Overall, these data indicate that maturation of the camel gut microbiome is accompanied by a reduction and focusing of the resistome and by a shift in the dominant types of MGEs. This study provides an age-stratified reference for ARG reservoirs and MGE-associated ARG mobility in camels studied under conditions with no recorded therapeutic antibiotic treatments and may be useful for future work on antimicrobial resistance in extensively managed livestock.

**IMPORTANCE:**

Antimicrobial resistance is often studied in animals heavily exposed to antibiotics, leaving a gap in our understanding of its natural development. Camels, rarely treated with antibiotics, offer a unique model. By comparing juvenile and adult gut microbiomes, we found that early-life communities are diverse, unstable, and rich in mobile resistance genes, while adult communities are more stable and carry fewer mobile elements. These findings establish a natural baseline for how resistance genes emerge and settle without drug pressure, providing critical insights for One Health strategies aimed at limiting the spread of resistance in livestock and wildlife.

## INTRODUCTION

Antimicrobial resistance (AMR) is a growing global threat to human and animal health (1). The misuse and overuse of antibiotics in human medicine, veterinary practice, and agriculture have accelerated the selection and spread of antimicrobial resistance genes (ARGs), many of which are carried on mobile genetic elements (MGEs) such as plasmids and transposons (2–4). These MGEs facilitate horizontal gene transfer (HGT), including between distantly related bacterial species, thereby contributing to the global dissemination of multidrug-resistant strains (5, 6). While the gut resistome of humans and intensively farmed livestock such as cattle (7) and poultry (8, 9) has been studied extensively, the role of host age in shaping resistome structure is still underexplored, particularly in systems with no recorded therapeutic antibiotic treatments.

The gastrointestinal microbiota (10) is crucial for host health, nutrient metabolism, and immune defense, forming a complex ecosystem shaped by host genetics (11), diet (12), environmental exposures (13), and antibiotic use (14). Among these factors, host age is a critical but often understudied driver of microbial composition and function. In various mammalian species, gut microbial succession occurs during development (15, 16). Neonatal microbiota typically show high inter-individual variability and a predominance of facultative anaerobes, with dynamic community shifts influenced by maternal transmission and early diet (17). In contrast, adult microbiomes are generally more stable, functionally specialized, and resilient to perturbations (18). These age-related transitions influence microbial diversity, metabolic potential, and the composition and distribution of ARGs (19), potentially via changes in community stability and horizontal gene transfer rates. However, the impact of these developmental transitions on the gut resistome, especially in systems with no recorded therapeutic antibiotic treatments, remains poorly characterized.

Camels, often referred to as the “ships of the desert,” are pseudo-ruminants with a three-chambered forestomach that enables efficient fermentation of low-quality, fibrous forage (20). Unlike intensively farmed cattle and pigs (21, 22), camels in extensive grazing systems are generally considered to experience no recorded therapeutic antibiotic treatments, making them an ecologically relevant model for studying the natural dynamics of ARGs. Camel gut microbiota are essential for fiber degradation, nutrient assimilation, and immune modulation, yet age-associated changes in their structure and function remain largely uncharacterized (23, 24). Recent studies have begun to describe camel gut microbiota and to detect ARGs in camel milk, but age-resolved gut resistome and mobilome data are still lacking. In other herbivores, including cattle (7), sheep, and goats (25), gut microbial diversity and metabolic capacity change substantially across developmental stages. Juveniles typically harbor more diverse and plastic microbiomes, whereas adults develop specialized consortia optimized for fiber digestion. Whether similar age-related microbial transitions occur in camels and how these changes influence the gut resistome and MGEs are unknown.

To address this gap, we classified dromedary camels into two age groups: juveniles (approximately 6 months old, representing an early-life developmental stage) and adults (6 years of age), based on commonly recognized developmental stages. Fecal samples from 18 juveniles and 15 adults were subjected to shotgun metagenomic sequencing, generating approximately 10 Gb of high-quality reads per sample. We analyzed age-related differences in the diversity, structure, and ecological stability of microbial communities. In addition, we profiled ARGs and characterized carbohydrate-active enzyme (CAZyme) repertoires and mobile genetic elements (MGEs). Because none of the enrolled animals had recorded antibiotic treatments according to farm records and veterinary reports, our study provides an age-resolved view of the camel gut microbiome, resistome, and mobilome under the study conditions and offers a reference framework for ARG reservoirs in extensively reared camels.

## MATERIALS AND METHODS

### Animal management and diet

All camels were kept under the same extensive grazing management on a pasture-based farm in Xinjiang, China. Juvenile (SM) and adult (SY) animals belonged to the same herd, shared the same paddocks, and grazed together daily on natural desert steppe vegetation dominated by camel thorn (locally known as “luotuoci”) and desert bunchgrasses (e.g., “jijicao”). During the winter season, both age groups received the same supplemental alfalfa hay to help maintain the body condition under low-temperature and low-pasture-availability conditions. These feeding conditions reflect typical management in this production system, with juveniles and adults exposed to the same grazing environment and winter supplementation regime but differing in their reliance on milk versus exclusively forage-based diets.

### Sample collection and DNA extraction

Fresh fecal samples were collected from healthy camels in two age groups (6 months and 6 years) under sterile conditions to minimize environmental contamination. According to farm management records and consultations with local veterinarians and animal owners, none of the enrolled camels received therapeutic or prophylactic antibiotic treatments during the study period. Routine in-feed or in-water antibiotic administration is not reported for the animals studied in this extensive grazing system.

During sampling, freshly defecated fecal pellets were collected, and samples with visible contact with soil or standing water were avoided to minimize environmental contamination. A negative extraction control was included during DNA extraction to monitor potential laboratory contamination. However, antibiotic residues in feed, drinking water, or the surrounding environment were not directly measured, and field blank controls were not included.

DNA integrity was assessed by 1% agarose gel electrophoresis. Purity (A260/A280 ≈ 1.8) and concentration (> 50 ng/µL) were measured using a NanoDrop 2000 spectrophotometer (Thermo Fisher Scientific, USA) and a Qubit 2.0 Fluorometer (Life Technologies, USA) with the dsDNA HS Assay Kit, respectively. Only high-quality DNA samples were used for library construction.

### Library preparation and sequencing

For each sample, approximately 500 ng of high-quality genomic DNA was used to construct shotgun metagenomic libraries using the TruSeq DNA PCR-Free Library Prep Kit (Illumina, San Diego, USA). DNA fragmentation was performed using a Covaris ME220 ultrasonicator (peak incident power 50 W; duty factor: 20%; treatment time: 55 s) to achieve an average insert size of approximately 350 bp. The fragmented DNA was subjected to end repair, A-tailing, and ligation with dual-index barcoded adapters for multiplexing.

Library quality and insert size distributions were assessed using an Agilent 2100 Bioanalyzer (Agilent Technologies, USA). Only libraries with the expected fragment profiles and sufficient concentration were selected for paired-end sequencing (2 × 150 bp) on the Illumina NovaSeq 6000 platform, generating approximately 10 Gb of raw data per sample.

### Metagenomic assembly and gene annotation

Host-depleted reads from each sample were *de novo-*assembled into contigs using MEGAHIT (v1.0.6) with default settings. Assembly quality was evaluated based on total contig length and N50 statistics. Open reading frames (ORFs) were predicted from assembled contigs using Prodigal (v2.6.3) in the metagenomic mode. Predicted protein sequences from all samples were combined and clustered using CD-HIT (v4.8.1) at a 95% identity threshold to create a nonredundant gene catalog. Representative sequences were retained for downstream functional annotation.

Functional annotation of the nonredundant gene catalog was performed using several complementary databases. Predicted proteins were aligned against the KEGG GENES database, with KEGG orthology (KO) identifiers and metabolic pathways retrieved using KEGG Mapper. Additionally, proteins were annotated with eggNOG v5.0 using eggNOG-mapper (v2), yielding orthologous group assignments, COG functional categories, and Gene Ontology (GO) terms.

CAZymes were identified using both sequence similarity and domain-specific approaches. DIAMOND was used for fast alignment against the CAZy database, while HMMER3 (v3.3) and dbCAN HMMdb v10 were used for sensitive detection based on hidden Markov models. Detected CAZyme domains were assigned to established families, including glycoside hydrolases (GHs), glycosyltransferases (GTs), polysaccharide lyases (PLs), carbohydrate esterases (CEs), and carbohydrate-binding modules (CBMs).

ARGs were identified using the Comprehensive Antibiotic Resistance Database (CARD, v3.2.6). In addition to a dedicated resistome profiling pipeline, DIAMOND-based alignment against CARD was performed to cross-validate the ARG calls. Significant hits were annotated with Antibiotic Resistance Ontology (ARO) terms and required database-specific thresholds (e.g., ≥30% identity and ≥70% alignment length). Annotations were based on the top-scoring alignment (highest bit score), although multiple annotations per gene were retained when relevant,for example, when a gene contained both enzymatic and resistance-related domains.

### Mobile genetic element annotation

MGEs were identified at the protein level using MobileOG-db. Predicted protein-coding genes in the nonredundant camel gut gene catalog were searched against MobileOG-db using DIAMOND blastp (v2.0.9) with an e-value threshold of 1e−5 and minimum alignment coverage and identity cutoffs consistent with those used for ARG annotation. For each gene, only the best hit that satisfied these thresholds was retained and assigned to the MGE type reported in the MobileOG-db annotation.

For the purposes of this study, MobileOG-db categories were collapsed into broader functional classes. “Transposases” comprised genes annotated as transposase or transposition-related proteins (e.g., Tn3-family transposases) that did not carry an insertion sequence (IS) family designation. “Insertion sequences (IS elements)” comprised genes annotated as IS element or IS-family transposase, or genes bearing canonical IS family names (e.g., IS3, IS30, ISL3, and IS110) in the MobileOG-db description. Proteins annotated as integrase, recombinase, relaxase, type IV secretion system components, or other conjugative transfer factors were grouped as “integrative/conjugative elements.” Unless otherwise stated, references to “transposases” and “IS elements” in the main text refer to these gene-level categories derived from MobileOG-db annotations.

### Statistical and bioinformatic analyses

All statistical analyses were conducted in R (v4.2.2). Microbial alpha diversity was evaluated using observed species richness, the Chao1 richness estimator, and the Shannon diversity index. Species-level taxonomic profiles, derived from Kraken2 classifications or MAG relative abundances, were rarefied to a uniform sequencing depth before calculation. Diversity indices were calculated using the vegan package (v2.6-4). Differences in alpha diversity between juvenile and adult groups were tested using the two-sided Wilcoxon rank-sum test due to the non-normal data distribution.

Beta diversity was assessed using Bray–Curtis dissimilarity, calculated from genus-level relative abundance tables. Principal coordinates analysis (PCoA) was used for ordination, and community compositional differences between groups were assessed using permutational multivariate analysis of variance with the adonis function in vegan.

To identify age-discriminatory taxa, linear discriminant analysis effect size (LEfSe; Galaxy v1.0) was used on genus-level relative abundance profiles. Co-occurrence networks were constructed to explore potential associations between microbial taxa and ARGs. Sparse correlations were estimated using SparCC. Genus-level microbial and ARG abundance tables were filtered to retain features present in ≥50% of samples, with a mean relative abundance ≥0.1%.

SparCC was run with 100 iterations for correlation inference and 500 bootstraps to estimate pseudo *P*-values. Correlation analyses focused on genus–ARG family pairs. Edges with SparCC *r* > 0.3 and FDR-adjusted *P* < 0.05 were considered robust and retained for downstream network construction.

Networks were visualized in Cytoscape (v3.9.1), where nodes represented either bacterial genera or ARG families, and edges denoted significant positive or negative associations. Topological parameters, including node degree and betweenness centrality, were calculated to identify network hubs. To assess age-dependent variation, separate co-occurrence networks were generated for juvenile and adult groups, and connectivity patterns were qualitatively compared. All computational analyses were performed on a Linux-based high-performance computing cluster.

## RESULTS

**Fig 1:**
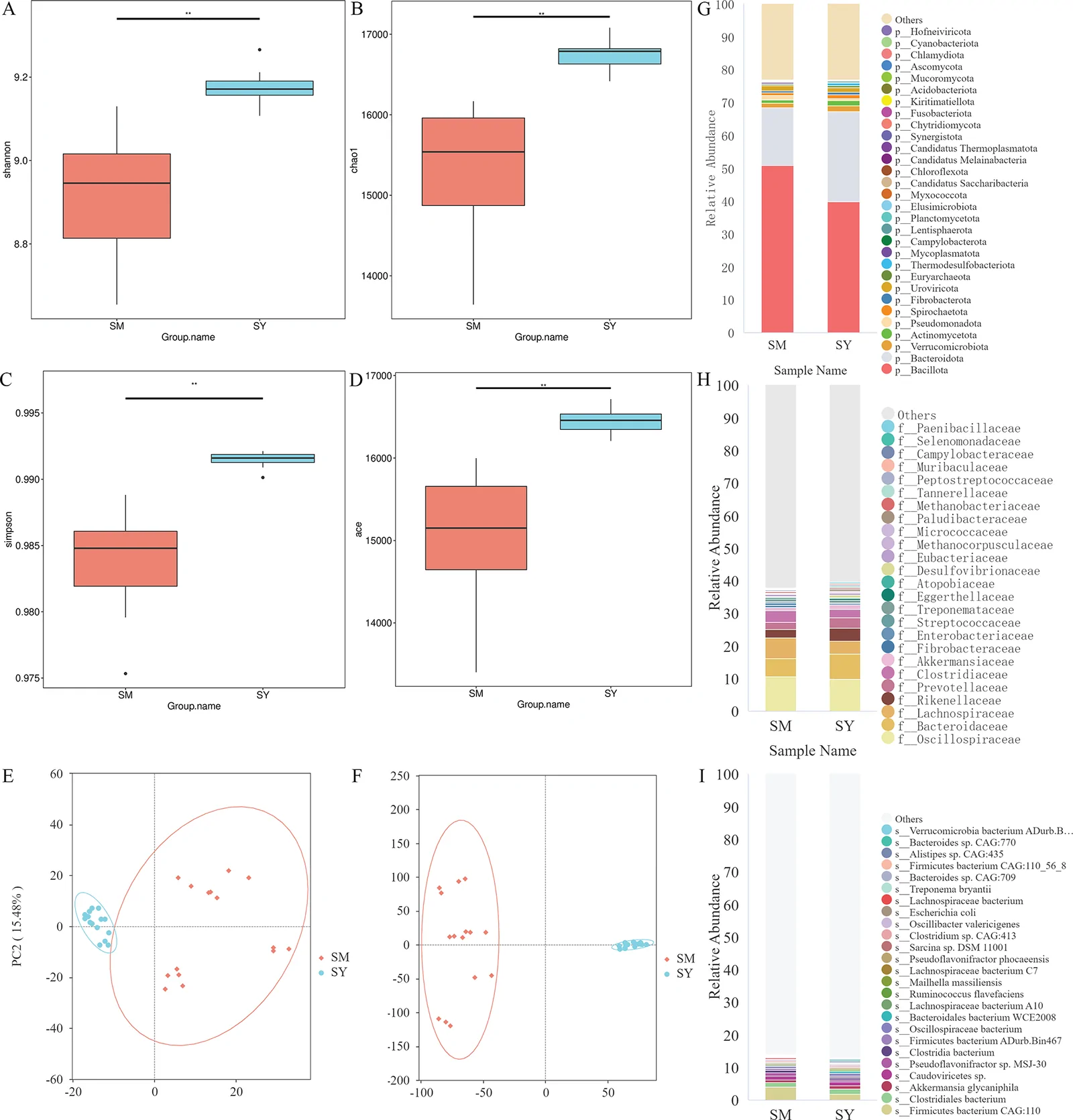
Differences in the intestinal microorganisms of camels at different stages. (**A–D**) Alpha diversity indices (Shannon, Chao, Simpson, and ACE) in the two groups. Comparisons between the SY and SM groups: *P* < 0.05, *P* < 0.01 (Kruskal–Wallis test with Dunn’s post hoc test). (**E and F**) PCoA of the Bray–Curtis distances between the microbiota at the family and species levels. (**G**) Bacterial abundances at the phylum level. (**H**) Bacterial abundances at the family level. (**I**) Bacterial abundances at the species level.

Fresh fecal samples were collected from healthy camels in two age groups (6 months and 6 years). Detailed metadata are provided in Table S1. Compared to juveniles, adults exhibited a significantly greater alpha diversity, with higher Shannon indices (4.23 ± 0.31 vs 3.51 ± 0.28, *P* < 0.01) and Pielou’s evenness (0.78 ± 0.05 vs 0.65 ± 0.06, *P* < 0.01) (Fig. 1A through D). PCoA based on Bray–Curtis dissimilarity further revealed clear compositional separation between the two age groups (Fig. 1E and F), highlighting a pronounced age-associated shift in the gut microbial community structure.

At the phylum level, Bacillota (formerly Firmicutes; 52.3%) and Bacteroidota (formerly Bacteroidetes; 38.1%) predominated in all samples. Juveniles harbored higher levels of Bacillota and Pseudomonadota (formerly Proteobacteria), phyla that include many facultative anaerobic taxa. In contrast, adults showed higher abundances of Bacteroidota (42.7%) and archaeal lineages such as Thermoplasmatota and Euryarchaeota, taxa commonly associated with complex carbohydrate fermentation. These taxonomic transitions, detailed in Fig. S1, likely reflect a developmental progression from oxygen-tolerant, fast-growing colonizers to obligate anaerobes specialized in fiber degradation, mirroring the host’s dietary shift and rumen-like foregut fermentation demands.

At finer taxonomic resolution, the families *Oscillospiraceae*, *Bacteroidaceae*, *Lachnospiraceae*, *Rikenellaceae*, and *Prevotellaceae* were consistently dominant in both groups (Fig. 1H and I). Notably, microbial communities in juveniles exhibited significantly higher inter-individual variability, consistent with a more heterogeneous gut community structure during early life.

**Fig 2:**
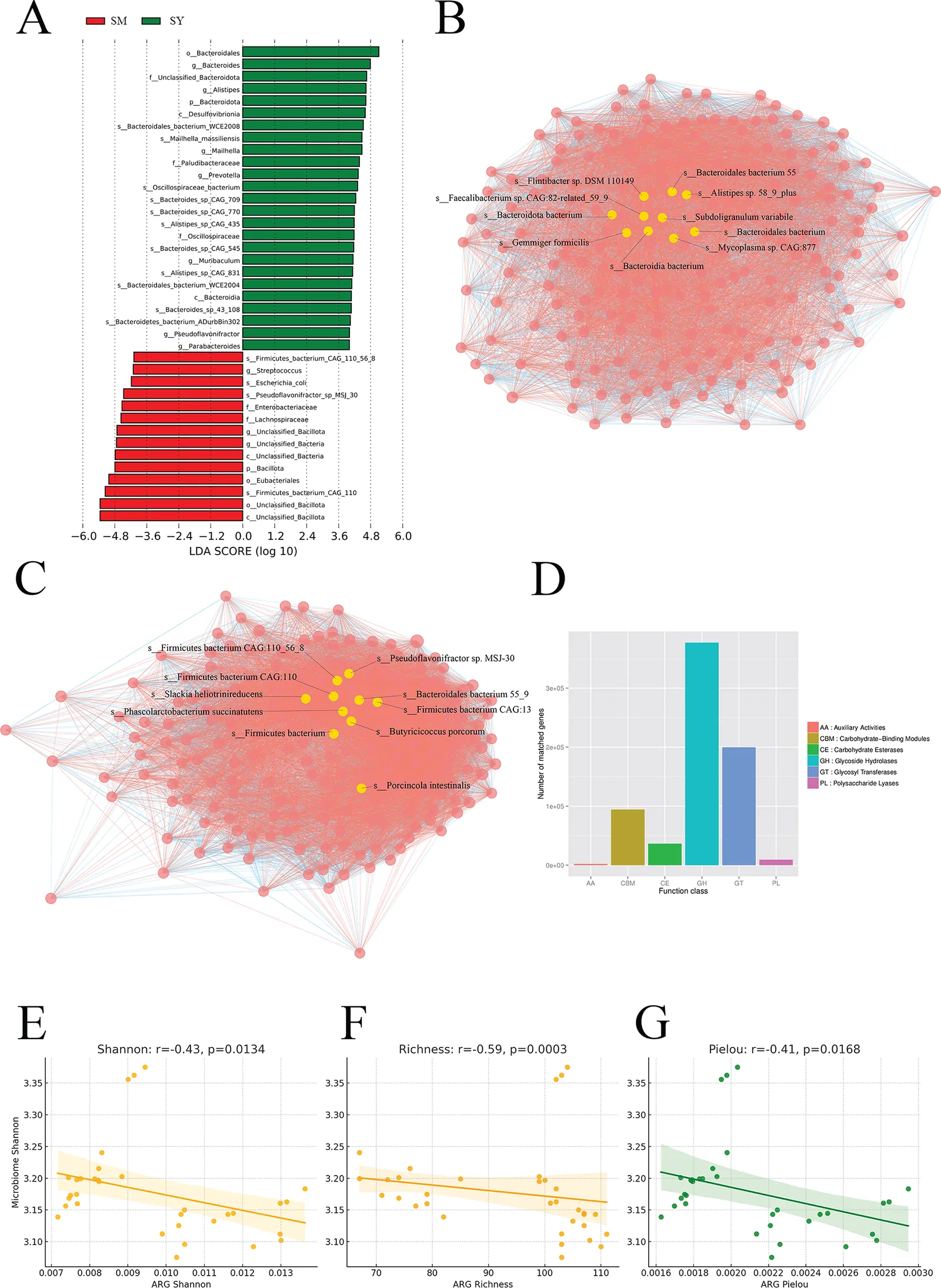
Differences in gut microbiota composition and ARGs between juvenile SM and adult SY camels. (**A**) Linear discriminant analysis effect size (LEfSe, LDA score > 3.0, *P* < 0.05) was used to identify differentially abundant bacterial taxa between groups. Taxa enriched in the SM group are shown in red, while those enriched in the SY group are shown in green. (**B and C**) Age-stratified co-occurrence networks were constructed based on SparCC. Correlations were computed among the top 50 bacterial genera, with robust associations defined as SparCC *r* > 0.3 and FDR-adjusted *P* < 0.05. Nodes represent genera, and edges indicate significant correlations. Node size and color reflect degree centrality. The top 10 genera with the highest centrality are highlighted in yellow. (**D**) Functional classification of significantly enriched bacterial taxa based on the CAZy database (annotated using dbCAN HMMdb v10). CAZyme categories are grouped by primary class, including GTs, GHs, and carbohydrate-binding modules (CBMs). The y-axis represents relative abundance. (**E–G**) Spearman’s rank correlation analyses were performed between ARG abundance and microbial diversity indices (Shannon, richness, and Pielou). All three correlations were negative, and all were statistically significant (Shannon: *r* = −0.43, *P* < 0.05; richness: *r* = −0.59, *P* < 0.05; Pielou: *r* = −0.41, *P* < 0.05). Each dot represents a sample. Shaded areas represent 95% confidence intervals.

The total number of edges in the SY group network was higher than that in the SM group, indicating slightly greater network complexity in adults than in juveniles (Fig. 2B). Centrality analysis revealed 18 core taxa (degree > 50) consistently present in both networks, primarily from Bacteroidaceae and Oscillospiraceae.

In the adult (SY) group, hub taxa such as Pseudoflavonifractor sp. MSJ-30, Phascolarctobacterium succinatutens, and Slackia heliotrinireducens likely contribute to maintaining functional coherence and ecological stability within the mature camel gut microbiome.

Metagenomic analysis revealed significant age-associated divergence in CAZyme profiles (Fig. 2D). Juvenile camel microbiomes were enriched in glycosyltransferases (GTs), which accounted for 41.2% of total CAZyme annotations, compared with 27.6% in adults (*P* < 0.01, Wilcoxon rank-sum test), consistent with glycan biosynthesis and modification during milk-dominated early development. In contrast, adult microbiomes were significantly enriched in glycoside hydrolases (GHs; 45.8% vs 31.4%, *P* < 0.01) and carbohydrate-binding modules (CBMs; 14.6% vs 7.3%, *P* < 0.05), reflecting microbial adaptation to a plant-based diet rich in complex polysaccharides. Adult communities also exhibited higher levels of cellulosome-associated components—including GH9, GH48, and dockerin/cohesin domains—indicating enhanced lignocellulose-degrading capacity. Collectively, these findings demonstrate an age-dependent functional transition from glycan synthesis to polysaccharide degradation, driven by the dietary shift from milk to fibrous forage and accompanied by ecological maturation of the camel gut microbiota.

### Resistome dynamics across developmental stages

A total of 2,724 ORFs annotated as ARGs were aligned with the NCBI NR protein database (version 2019-04) using BLASTp, with an E-value threshold of 1e–5 to assign putative bacterial hosts. Using metagenomic sequencing data from camel fecal samples, we assessed resistome diversity and its relationship with gut microbial composition across juvenile and adult groups.

Correlation analyses revealed consistent negative associations between ARG profiles and microbiome diversity indices. Specifically, the Shannon index of the resistome showed a moderate negative correlation with microbiome Shannon diversity (*r* = –0.43, *P* = 0.0134; Fig. 2E), indicating that samples with more diverse and evenly distributed ARGs tended to harbor less diverse bacterial communities. ARG richness also correlated negatively with microbial richness (*r* = –0.59, *P* = 0.0003; Fig. 2F), indicating that a broader repertoire of resistance genes was linked to reduced taxonomic complexity. A similar pattern was observed for Pielou’s evenness (*r* = –0.41, *P* = 0.0168; Fig. 2G), where higher evenness in ARG abundance was associated with decreased species evenness in the gut microbiota.

These findings suggest a potential ecological trade-off between the expansion of antimicrobial resistance and the maintenance of microbiome diversity. Similar inverse relationships have been reported in humans and livestock, where ARG enrichment is often associated with reduced microbial complexity or dysbiosis under selective pressure (26). Such patterns may reflect competitive exclusion by ARG-harboring taxa or functional redundancy within the resistome, limiting ecological space for diverse microbial communities.

**Fig 3:**
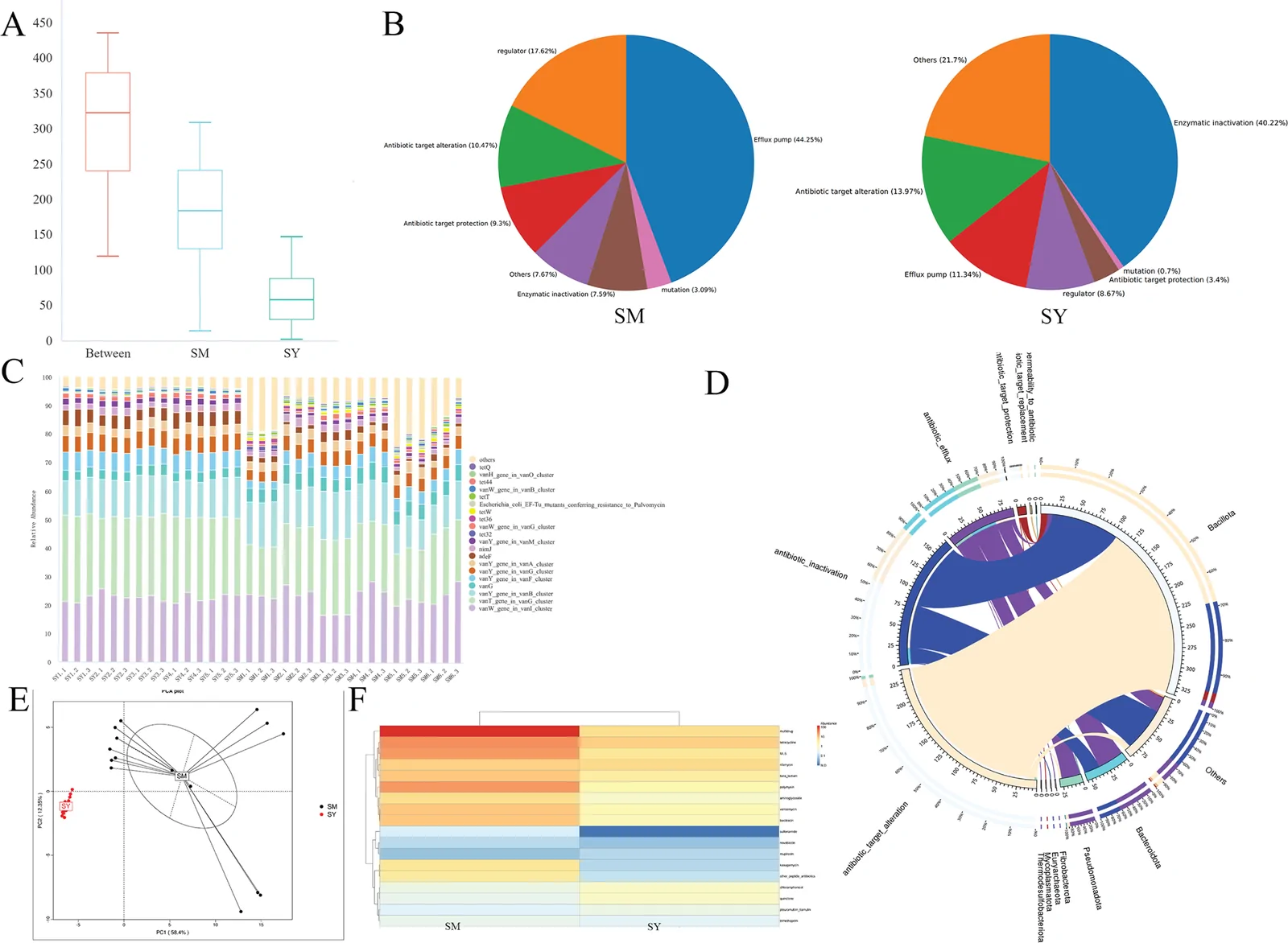
Temporal dynamics of gut resistome structure and composition across the developmental stages of camels. (**A**) The total number of ARGs was significantly higher in juvenile camels than in adults, suggesting greater resistome richness in early life. (**B**) Distribution of ARG mechanisms is represented by pie charts. The SM group was dominated by antibiotic efflux (44.25%), while the SY group exhibited increased enzymatic inactivation (40.22%). (**C**) Relative abundance of ARG categories: vanW and vanY were enriched in the SM group, while tetQ and tetW predominated in the SY group. (**D**) Chord diagram illustrating the associations between resistance mechanisms and bacterial phyla. (**E**) PCoA based on Bray–Curtis dissimilarity demonstrated greater inter-individual resistome variability in SM samples compared to the more uniform SY group. (**F**) Heatmap showing the distribution of major ARG categories by abundance. SM camels exhibited higher levels of multidrug, tetracycline, and macrolide–lincosamide–streptogramin (MLS) resistance, while the SY group retained only core ARG classes at lower abundance.

A total of 118 unique ARGs were identified across all samples, covering 35 resistance classes. ARG richness and alpha diversity were significantly higher in juvenile camels (SM group), with an average of 47.6 ± 5.2 ARGs per sample, compared to 32.1 ± 4.7 in adults (SY group) (*P* < 0.01; Fig. 3A). This suggests a more complex and diverse resistome in early life stages.

Analysis of resistance mechanisms revealed distinct age-related patterns. In the SM group, antibiotic efflux was the predominant mechanism, accounting for 44.25% of all annotated ARGs. This suggests that microbial communities in young camels primarily rely on energy-dependent transport systems to expel antibiotics, thereby reducing intracellular concentrations. Efflux-dominated profiles are frequently associated with fast-growing, generalist taxa and may reflect a transitional microbial ecology.

In contrast, adult camels exhibited a marked shift toward enzyme-mediated antibiotic inactivation, constituting 40.22% of all ARGs (Fig. 3B). This mechanism included functional classes such as β-lactamases and aminoglycoside-modifying enzymes, which chemically degrade or alter antibiotics to neutralize their activity. Furthermore, mechanisms related to target modification and antibiotic target protection were significantly more abundant in the SY group, comprising 21.7% of resistance pathways compared to 7.67% in juveniles. These mechanisms often involve structural changes to ribosomal subunits, DNA gyrases, or peptidoglycan synthesis enzymes, conferring high-level resistance without metabolic cost.

These findings collectively highlight an age-dependent shift in ARG composition and resistance strategies—from broad-spectrum, efflux-based defenses in juveniles to enzymatic and target-level adaptations in adults—reflecting the microbial maturation and ecological stabilization of the camel gut environment.

### ARG cluster distribution across groups

The distribution of ARG clusters differed significantly between juvenile (SM) and adult (SY) camels (Fig. 3C). Young camels exhibited higher abundances of vancomycin resistance genes, particularly vanW and vanY, while adults were dominated by tetracycline resistance genes such as tetQ and tetW. These patterns are consistent with age-associated differences in resistome composition, with juveniles harboring a broader repertoire and adults exhibiting more focused resistance profiles. Although less diverse overall, the adult resistomes showed greater compositional consistency and were compositionally consistent than those of juveniles.

Taxonomic profiling of resistance mechanisms revealed phylum-level associations: antibiotic inactivation genes were primarily associated with Bacteroidota and Pseudomonadota, while target alteration and efflux were mainly mediated by Bacillota and Pseudomonadota (Fig. 3D). Heatmap analysis (Fig. 3F) confirmed higher abundances of multidrug, tetracycline, and macrolide–lincosamide–streptogramin (MLS) resistance genes in the SM group, along with moderate levels of β-lactam and aminoglycoside resistance. Relative to the SM group, the SY group exhibited reduced resistome complexity, mainly retaining core resistance classes—such as tetracycline—at lower relative abundances.

Intra-group variation also differed by age: SM samples exhibited pronounced inter-individual variability in ARG profiles, while SY resistomes clustered tightly (Fig. 3E), suggesting greater ecological stability in the mature gut microbiome.

Taken together, these findings reveal a developmental shift from broad, efflux-dominated resistance profiles in juveniles to functionally streamlined enzymatic strategies in adults, paralleling microbiome maturation and ecological adaptation in the camel gastrointestinal tract.

### Bacterial species carrying ARGs

**Fig 4:**
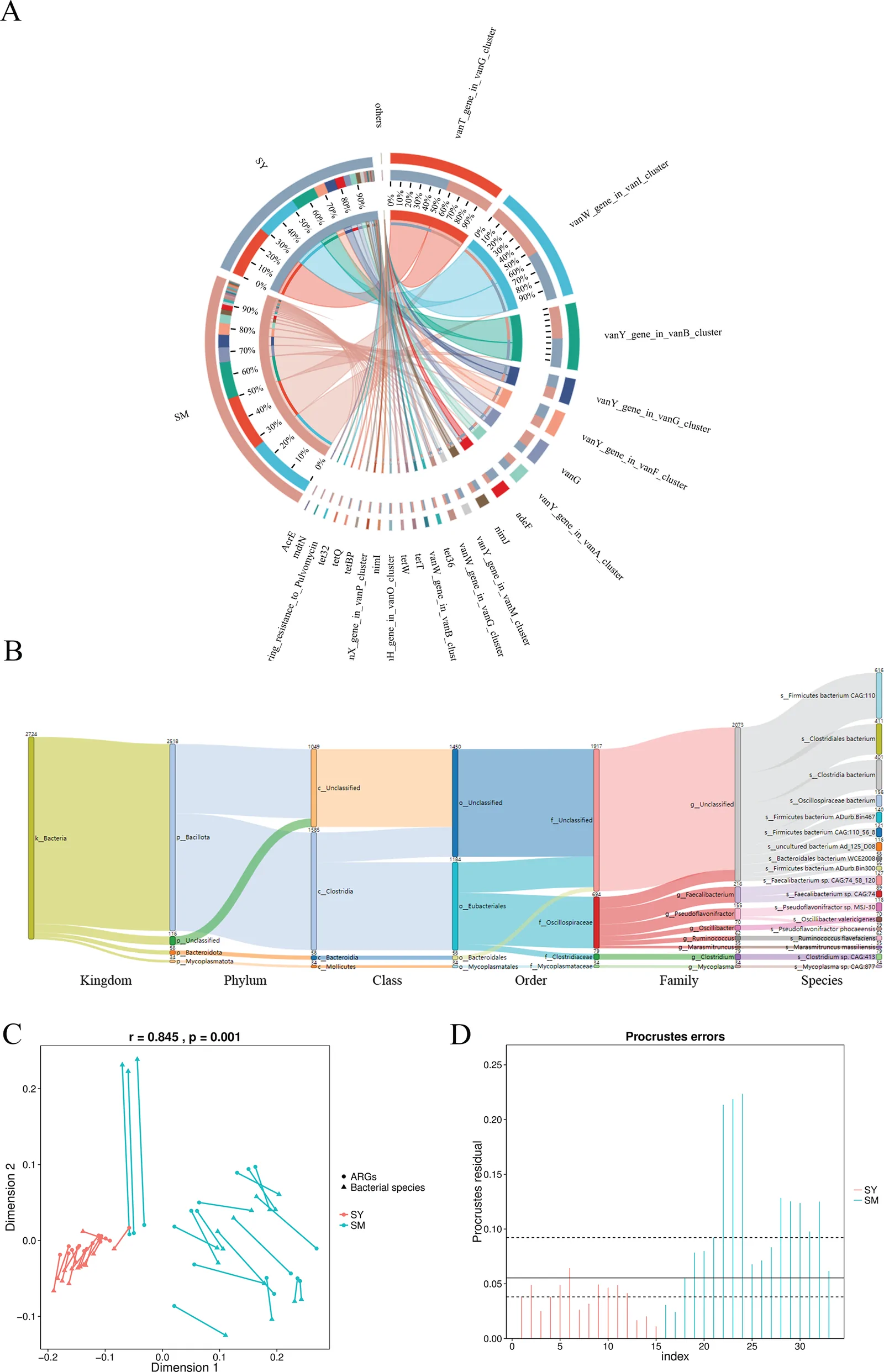
Structure and taxonomic distribution of the camel fecal resistome across developmental stages. (**A**) Relative abundances of ARGs in individual samples from juvenile (SM) and adult (SY) camels. Each stacked bar represents the proportional composition of ARGs per sample. (**B**) Taxonomic assignment of 2,724 ARG-annotated protein-coding genes across bacterial lineages. The length of the rectangle reflects the number of ARGs, and color denotes taxonomic rank. The most prominent ARG contributor was *Firmicutes bacterium* CAG:110, represented by the largest and darkest-colored rectangle. Sankey diagram showing the taxonomic distribution of all 2,724 ARG-annotated protein-coding genes from kingdom to species. Each vertical bar corresponds to a taxonomic rank (kingdom, phylum, class, order, family, and species), and each colored segment within a bar represents a taxon at that rank. The height of segments and the width of ribbons connecting ranks are proportional to the number of ARG-annotated genes assigned to that taxon, so thicker flows indicate larger ARG reservoirs. Colors distinguish bacterial phyla (e.g., Bacillota, Bacteroidota, Pseudomonadota [formerly Proteobacteria], Actinobacteriota, Mycoplasmatota, and unclassified taxa), as indicated in the accompanying color key. Numbers above each bar denote the total number of ARG-annotated genes at that rank. (**C**) Ordination-based comparison of bacterial species and ARG profiles through Procrustes analysis. Line lengths between paired points (microbial vs ARG coordinates) correspond to Procrustes residuals, with longer distances indicating greater divergence. (**D**) Summary of Procrustes residuals for all samples.

ARGs were widely distributed across bacterial taxa, with a marked concentration in Bacillota, particularly within the class Clostridia. Key ARG-harboring orders included *Clostridiales*, *Bacteroidales*, and *Pseudomonadales*. Notably, *Firmicutes bacterium* CAG:110 (a species name retained as in the NCBI taxonomy), a taxon affiliated with the phylum Bacillota under current nomenclature, carried the highest number of resistance genes (616 out of 2,724 ARG-annotated ORFs; Fig. 4B) and was visualized as the largest and darkest-colored node in the network plot, underscoring its role as a dominant ARG reservoir in the camel gut microbiome.

To assess the structural congruence between microbial community composition and resistome profiles, Procrustes analysis was used. This multivariate ordination technique compares two datasets by superimposing their principal coordinates, quantifying their alignment across samples. Figure 4C shows Procrustes residuals for individual samples: smaller residuals indicate close alignment between bacterial species distributions and ARG profiles, while larger residuals reflect structural divergence.

Figure 4D summarizes the overall distribution of residuals using the median (solid line) and interquartile ranges (dashed lines), showing that most samples exhibited moderate and consistent alignment. However, several samples deviated significantly, suggesting sample-specific decoupling between taxonomic structure and resistome composition.

Taken together, these results suggest that while microbiota and resistome compositions are generally well-aligned in the camel gut, distinct inter-sample heterogeneity persists, highlighting both structural stability and context-specific divergence in host–ARG associations.

### Age-related differences in ARG dynamics and MGE-mediated dissemination pathways

Metastat analysis revealed significant age-related differences in ARG composition (Fig. S2). Juvenile camels (SM group) harbored significantly higher levels of vancomycin resistance genes (vanW, vanT, and vanY) and tetracycline resistance genes (tet32, tetW, and tet36), while the multidrug efflux gene adeF was reduced relative to the adult group (SY).

**Fig 5:**
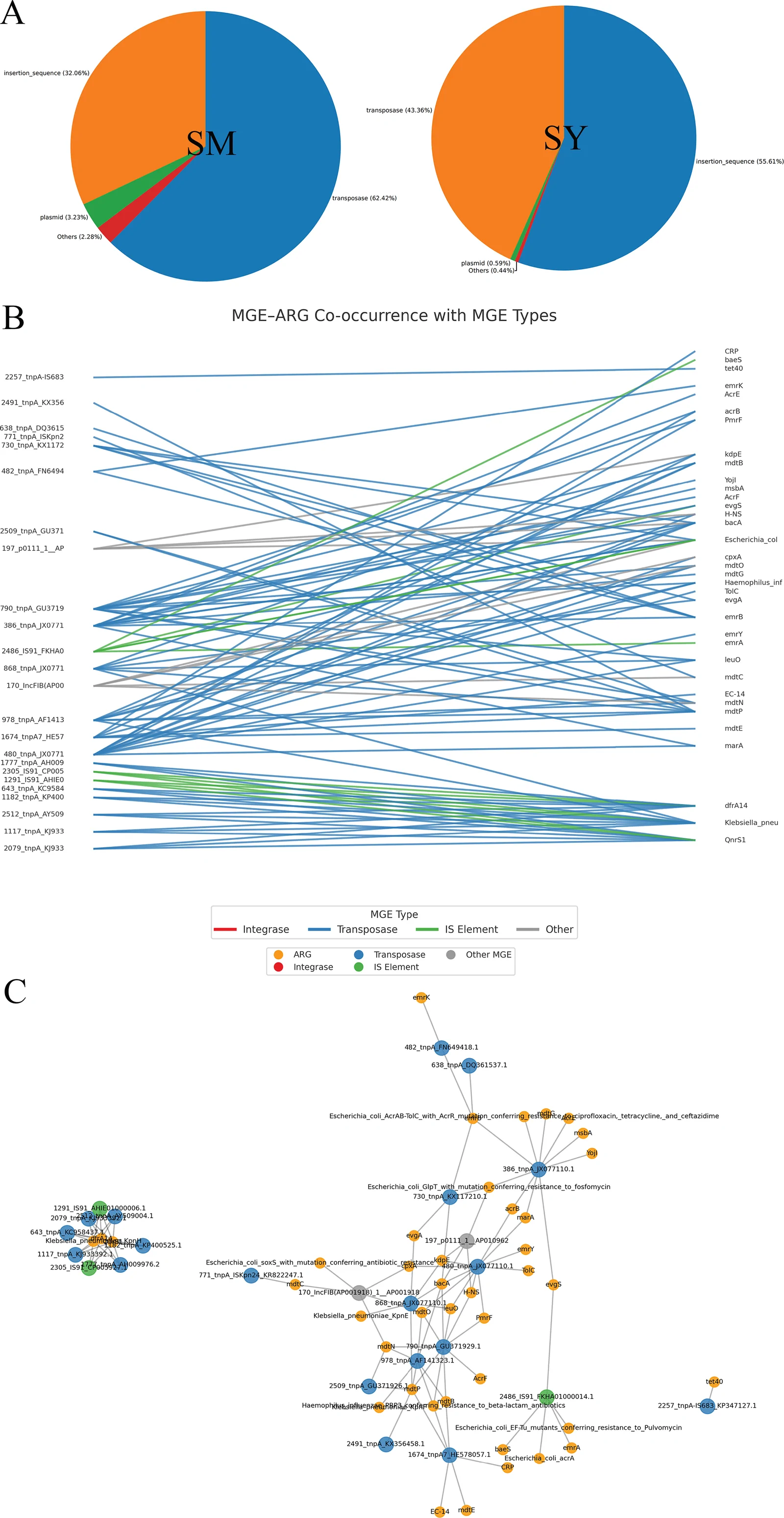
Association patterns between ARGs and MGEs across camel gut samples. (**A**) Relative contribution of different MGE types carrying ARGs in juvenile (SM) and adult (SY) groups. Pie charts show the proportions of transposases, IS elements, plasmid-associated MGEs, and other MGEs among all ARG-carrying MGEs in each age group. (**B**) Significant co-occurrence relationships between MGEs (left) and ARGs (right). Each line represents a significant MGE–ARG association (SparCC *r* ≥ 0.3, *P* < 0.05) and is colored by MGE type (red, integrase; blue, transposase; green, IS element; gray, other MGEs). (**C**) Co-occurrence network highlighting clusters of MGEs and ARGs. Nodes represent ARGs (orange) or MGEs (red, integrase; blue, transposase; green, IS element; gray, other MGEs). Edges indicate significant co-occurrence links (same thresholds as in panel B), and the layout is based on a force-directed algorithm to emphasize hub ARGs and MGEs.

MGEs also exhibited age-specific signatures (Fig. 5A). In juveniles, transposases predominated (62.42%), possibly reflecting dietary transitions and increased microbial plasticity during early development. In contrast, insertion sequences (IS elements) dominated the adult group, consistent with increased genomic stability. Plasmid-associated sequences were rare in both groups, suggesting limited potential for conjugative transfer.

To investigate ARG mobilization pathways, a co-occurrence matrix was created to identify statistically significant MGE–ARG pairs. MGEs were annotated using MobileOG-db and classified into integrases, transposases, insertion sequences, and others. These associations were visualized, highlighting the connectivity between ARGs and their potential carriers.

Most co-occurrence links involved transposases (blue lines), suggesting that transposable elements are a major route for ARG mobility in the camel gut microbiome. Specific resistance genes, such as acrB, emrB, and qnrS1, were associated with IS elements (green lines), implicating IS-mediated transposition in ARG dissemination. Efflux pump genes and regulatory components frequently co-occurred with integrase-type MGEs (red lines), potentially indicating involvement in integron- or resistance island-associated structures.

To investigate codissemination patterns between MGEs and ARGs, we constructed a co-occurrence network based on the top 100 significant MGE–ARG associations (Fig. 5C). These associations were filtered using SparCC correlation (*r* > 0.3, *P* < 0.05), ensuring statistical robustness. Nodes represent ARGs and MGEs, color-coded by MGE type: transposases (blue), insertion sequences (green), integrases (red), and other MGEs (gray), while node size reflects degree centrality. Edges denote significant co-occurrence links.

Several tightly connected modules were identified, particularly those centered around transposase elements. These modules formed extensive networks with multiple ARGs, suggesting that transposases are key facilitators of horizontal gene transfer (HGT) in the juvenile gut microbiome. In contrast, insertion sequences formed more localized clusters, consistent with their role in site-specific insertion and genomic stabilization.

Centrality analysis identified tnpA_JX0771 and ISKpn2 as prominent “genetic hubs,” displaying high degree centrality and linking to a broad array of resistance determinants. Notably, several efflux pump genes (acrB and emrB) were significantly associated with both integrases and IS elements, indicating their mobility through multiple pathways and possible involvement in integron-mediated gene cassettes.

Collectively, these findings illustrate distinct age-related mobilome strategies: the SM resistome exhibits a modular architecture dominated by transposase-driven gene flow, reflecting rapid microbial adaptation during early-life transitions. In contrast, the SY group relies more heavily on IS-mediated integration, consistent with a stabilized adult microbiome with reduced genomic plasticity. The sparse presence of plasmid elements across both groups further supports the conclusion that ARG dissemination in camel guts is predominantly nonconjugative, shaped by intrinsic ecological succession rather than anthropogenic antibiotic pressure.

### Co-selection among antimicrobial resistance genes

**Fig 6:**
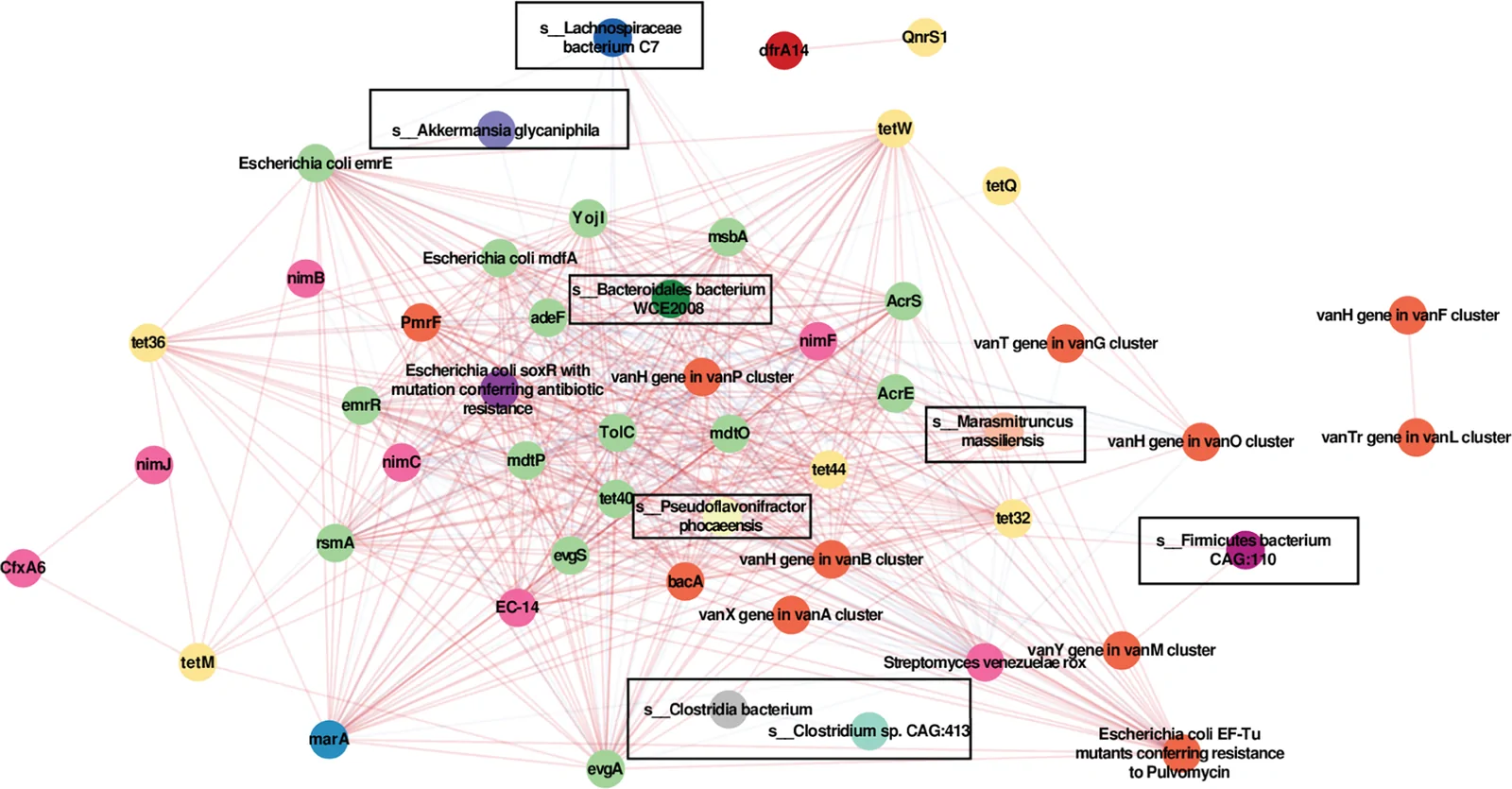
Co-selection network among prevalent ARGs in camel fecal samples. A correlation-based network was constructed using 50 ARGs detected in at least 50% of samples. Nodes represent individual ARGs, and edges denote significant positive correlations (SparCC *r* > 0.3, *P* < 0.05). Three main clusters indicate putative co-selection modules, with hub ARGs showing high degree centrality.

To evaluate potential co-selection among ARGs, we analyzed 50 ARGs detected in at least half of the metagenomically sequenced fecal samples. These genes were used to construct a correlation-based network to infer co-occurrence patterns (Fig. 6). The resulting network comprised 50 nodes, forming three distinct clusters, each representing a putative co-selection module.

Several ARGs exhibited strong positive associations with multiple other genes, suggesting potential co-mobilization or shared regulatory pathways. For instance, soxR from *Escherichia coli*, known to confer resistance via mutation-driven oxidative stress regulation, clustered tightly with other multidrug resistance genes. Likewise, vanH, part of the vanP operon, was co-associated with genes from disparate resistance classes, indicating its possible involvement in broader resistance cassettes.

The analysis also revealed ARGs within the genomes of bacterial taxa often considered beneficial, including Akkermansia glycaniphila and Lachnospiraceae bacterium C7. These findings raise important questions about the role of commensal or probiotic taxa as potential reservoirs of resistance genes and their contribution to resistome complexity within the gut ecosystem.

## DISCUSSION

We investigated how the gut microbiota, resistome, and mobile genetic elements change with age in extensively reared dromedary camels with no recorded therapeutic antibiotic treatments under the study conditions. Juvenile camels carried a broader and more complex resistome than adults (118 ARGs belonging to 35 resistance classes). With age, this expanded juvenile resistome contracted, and ARGs were more often embedded in chromosomal regions rather than being associated with highly mobile elements. These age-related changes in diversity and genetic context of ARGs are the main findings of this study. They are consistent with an association between gut microbiome maturation and reduced diversity and mobility of ARGs under the study conditions.

Our work also fills an important gap in camel microbiome research. Previous studies on dromedary and Bactrian camels mainly described taxonomic profiles in relation to diet, geography, and husbandry conditions. Only a few reports have linked fecal microbiota to specific ARG classes or documented ARGs in milk and other matrices, and most did not stratify animals by age or analyze the microbiome, resistome, and mobilome together (27–29). By jointly profiling these three layers in juvenile and adult camels, we show that microbiome maturation toward communities dominated by Bacteroidota and Bacillota is accompanied by a narrowing of the resistome, which in adults is dominated by β-lactamase and tetracycline resistance genes, and by a reconfiguration of the mobile gene pool. These findings complement reports of age-associated resistome declines in humans, pigs, and other mammals (30) and extend them to extensively managed livestock systems operating under comparable management conditions.

The observed contraction of the resistome with age likely reflects changes in gut ecology and microbial physiology. Juvenile camels showed higher abundances of opportunistic, fast-growing facultative anaerobes, including ARG-rich taxa such as *Escherichia–Shigella* and *Firmicutes bacterium* CAG:110, a taxon affiliated with the phylum Bacillota under the current nomenclature. These organisms are well adapted to relatively oxygen-rich, nutrient-variable conditions and often carry large multidrug resistance cassettes. As camels transition to a fiber-rich diet and the forestomach matures, obligate anaerobes such as *Prevotella*, *Rikenellaceae*, and *Alistipes*, which specialize in fiber degradation and typically harbor fewer ARGs, become dominant. This ecological succession toward consortia adapted to the host and specialized in fiber degradation (25) reduces ecological niches for ARG-rich generalists and may contribute to reduced opportunities for horizontal gene transfer, potentially stabilizing and contracting the resistome over time.

Microbiome–resistome links in our data set were not fully captured by α-diversity metrics alone. In juveniles, high ARG richness was associated with communities in which a few ARG-dense species dominated and evenness was low. This pattern suggests that taxonomic imbalance, rather than diversity *per se*, can favor resistome expansion. Adult camels, in contrast, had more even communities in which ARGs were distributed across several fiber-degrading taxa, and overall ARG richness and network connectivity were lower. These findings are consistent with the idea that microbiome evenness and functional redundancy, rather than species richness alone, may play an important role in constraining resistome expansion.

We also found clear age-related differences in resistance mechanisms and their genetic context. Juvenile camels harbored a broad spectrum of ARGs, including multidrug efflux pumps, vancomycin resistance determinants, and genes conferring co-resistance to metals and biocides, many of which are regarded as highly co-selectable (31). In the present study, however, the enrichment of van-associated genes in juveniles occurred in parallel with a resistome architecture strongly coupled to transposase-dominated mobile genetic elements. This pattern suggests that vanW/vanY enrichment is more parsimoniously interpreted as part of enhanced genome plasticity and horizontal gene transfer during early-life microbiome assembly, rather than being primarily driven by selection from a specific antimicrobial compound or feed additive. Although indirect co-selection by unmeasured environmental factors cannot be entirely excluded, our data indicate that age-associated differences in mobilome structure are consistent with an interpretation for the observed van gene patterns. Adults were enriched for genes encoding enzymatic inactivation, especially β-lactamases, together with a smaller set of tetracycline resistance genes (such as tetW and tetQ). In parallel, the mobilome shifted from juvenile networks dominated by transposase genes and other insertion sequences that strongly co-occurred with ARGs toward adult networks in which insertion sequences were proportionally more abundant, but plasmid signatures were rare, and ARGs were more frequently embedded in predicted chromosomal regions. These results indicate a transition from a highly mobile, broad-spectrum resistome in early life to a more integrated and specialized resistome in adulthood, with reduced potential for co-selection.

The strong coupling between ARG-rich opportunistic taxa and transposase-dominated mobile elements in juveniles provides clues about the origin and early assembly of the camel gut resistome. It suggests recent acquisition of resistance determinants via horizontal gene transfer, possibly amplified by maternal seeding from the dam’s gut and milk microbiota and by perinatal exposures (4, 30). Environmental microbiomes associated with water, feed, bedding, and human handlers (5, 26) are additional plausible sources. The presence of a diverse and highly mobile juvenile resistome in camels raised under extensive grazing management with no recorded therapeutic antibiotic treatments highlights the potential role of nontherapeutic selection pressures and environmental reservoirs in AMR dynamics in pastoral systems and has clear One Health relevance.

From a management perspective, our findings imply that promoting timely maturation of the gut microbiome may help accelerate resistome contraction in extensively reared camels. Approaches could include appropriate nutrition, avoiding overcrowding, and limiting avoidable environmental contamination. More broadly, our data show that developmental processes can substantially reduce the diversity and mobility of ARGs even under conditions of no recorded therapeutic antibiotic treatments. This pattern may serve as an ecological benchmark when evaluating interventions designed to minimize AMR in ruminant livestock.

This study has several limitations. First, we used a cross-sectional design and compared juvenile and adult camels under field conditions rather than following individuals over time. The age-related differences we describe, therefore, reflect contrasts between cohorts, not fully resolved developmental trajectories. However, the consistent differences observed in microbiome composition, resistome size, and mobile genetic element architecture support the conclusion that major restructuring occurs between early life and maturity. Second, although farm management records and veterinary reports indicated no recorded therapeutic antibiotic treatments or medicated feed use during the study period, antibiotic residues in feed, drinking water, or the surrounding environment were not directly measured, and field blank controls were not included during sampling. Consequently, low-level or unrecorded environmental exposure to antibiotics cannot be completely excluded. Finally, metagenomic sequencing and database-based annotation may underestimate rare or novel ARGs and mobile genetic elements, and we did not assess gene expression or *in vivo* resistance phenotypes. Future work incorporating sub-adult age classes, longitudinal dam-offspring cohorts, functional assays, and more extensive environmental sampling will be necessary to further refine the timing, mechanisms, and external drivers of resistome assembly and stabilization in extensively managed camel populations.

### Conclusion

This study offers a comprehensive metagenomic analysis of age-resolved changes in the camel gut microbiome, resistome, and MGEs under conditions of no recorded therapeutic antibiotic treatments. We found that juvenile camels harbor a diverse and flexible microbial community enriched with opportunistic taxa, broad-spectrum ARGs, and highly mobile genetic elements, especially transposases, reflecting a dynamic early-life environment. In contrast, adult camels possess a functionally specialized and compositionally stable microbiota. Their resistome is streamlined and dominated by core resistance genes and insertion sequences, consistent with a lower potential for horizontal gene transfer. These coordinated changes imply that microbiome maturation plays a key role in shaping the structure and mobility of the resistome via ecological filtering and decreased gene flow. By establishing a natural baseline for ARG distribution and mobility in camels with no recorded therapeutic antibiotic treatments during the study period, our findings offer valuable insights into the intrinsic development of antimicrobial resistance and serve as a reference for future One Health surveillance in wildlife and extensively managed livestock. More broadly, this work highlights the potential relevance of age-targeted approaches aimed at stabilizing the early-life microbiome for limiting the spread and amplification of resistance genes in animal hosts.

## Supplementary Material

Reviewer comments

## Data Availability

All raw sequencing data that support the findings of this study have been deposited in the Sequence Read Archive under the accession number PRJNA1256226.

## SUPPLEMENTAL MATERIAL

Supplemental figures spectrum.03183-25-s0001.docx Figures S1 and S2.

Supplemental tables spectrum.03183-25-s0002.xlsx Tables S1 and S2.

OPEN PEER REVIEW reviewer-comments.pdf An accounting of the reviewer comments and feedback.

ASM does not own the copyrights to Supplemental Material that may be linked to, or accessed through, an article. The authors have granted ASM a non-exclusive, world-wide license to publish the Supplemental Material files. Please contact the corresponding author directly for reuse.
