# Supplementary material for: Age-driven shifts of the camel gut microbiome and resistome in extensively reared dromedary camels
Source: Microbiol Spectr. 2026 Jun 3;14(7):e03183-25. doi: 10.1128/spectrum.03183-25 (PMC13340045; doi:10.1128/spectrum.03183-25)

**Age-Driven Shift of the Camel Gut Microbiome and Resistome in an Antibiotic-Free Environment**

Figure S1. Differences between the SM and SYgroups at the phylum level


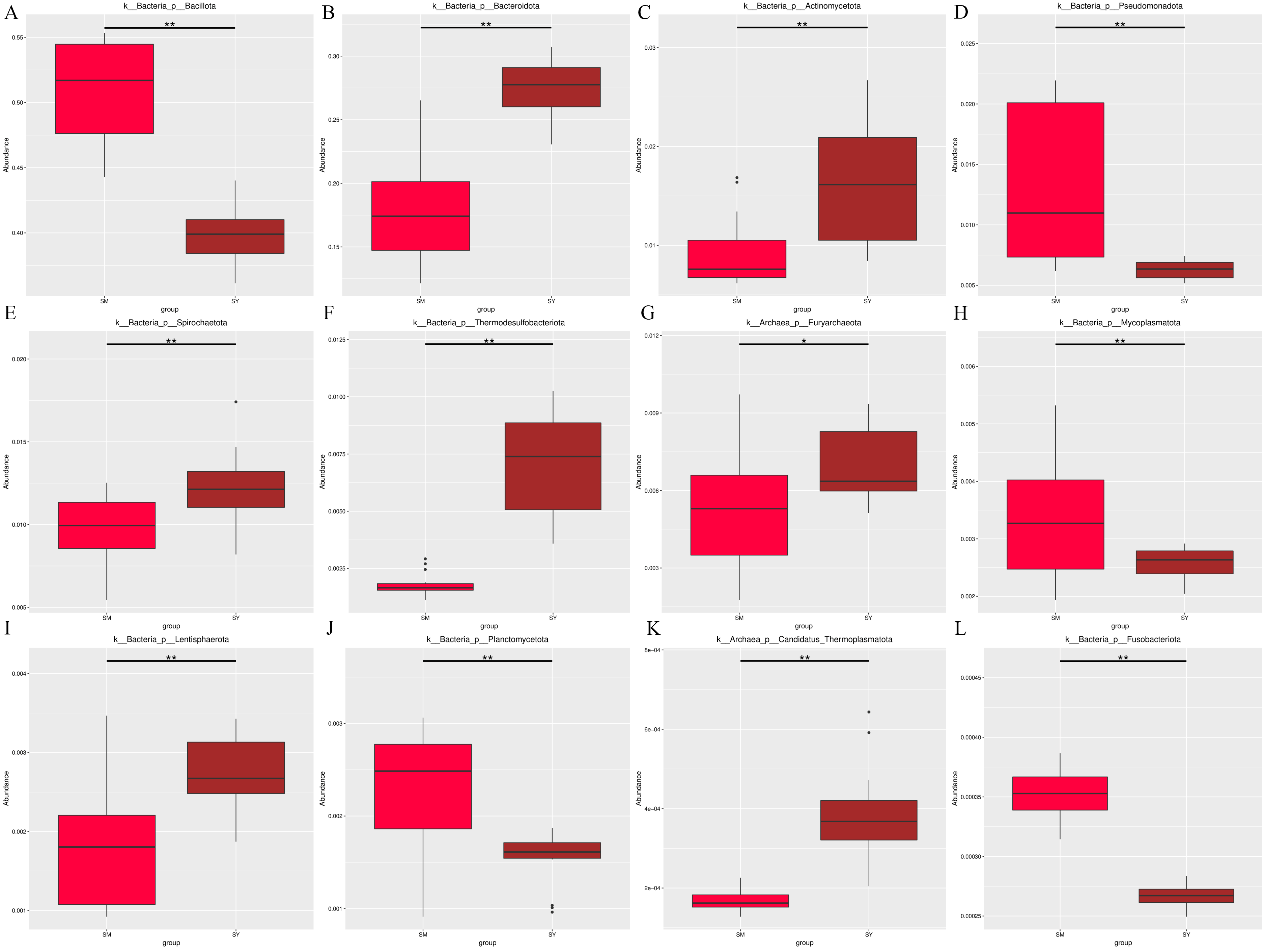


FigureS2. Differences between the SM and SY groups of the ARGs This result suggested that most of the ARGs might be constitutive.


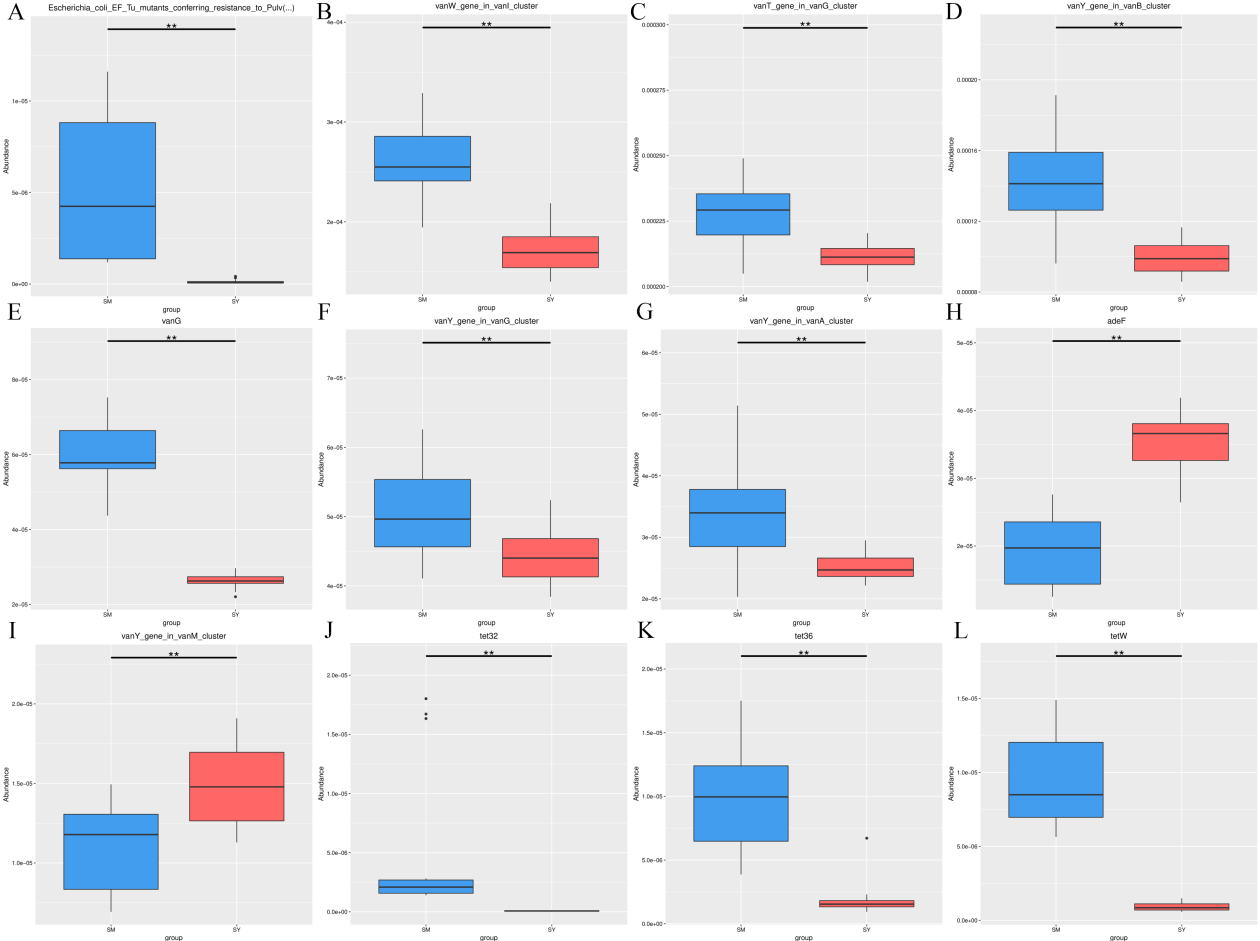

Supplement: Supplemental figures — Figures S1 and S2. [file spectrum.03183-25-s0001.docx]
