# Supplementary material for: Age-driven shifts of the camel gut microbiome and resistome in extensively reared dromedary camels
Source: Microbiol Spectr. 2026 Jun 3;14(7):e03183-25. doi: 10.1128/spectrum.03183-25 (PMC13340045; doi:10.1128/spectrum.03183-25)
Supplement: Reviewer comments [file reviewer-comments.pdf]

AMERICAN SOCIETY FOR MICROBIOLOGY | Microbiology Spectrum

Age-Driven Shifts of the Camel Gut Microbiome and Resistome in Extensively Reared Dromedary Camels

wenjing wang, yitao li, yaqian liang, junkai wang, zhihao zhang, yan zhang, chencheng xiao, and Haihong Hao

Corresponding Author(s): chencheng xiao, Shihezi University

Review Timeline:

Submission Date:	October 3, 2025
Editorial Decision:	December 2, 2025
Revision Received:	December 16, 2025
Editorial Decision:	January 12, 2026
Revision Received:	January 14, 2026
Accepted:	January 20, 2026

Editor: Hao-Yu Liu

Reviewer(s): The reviewers have opted to remain anonymous.

Transaction Report:

(Note: With the exception of the correction of typographical or spelling errors that could be a source of ambiguity, letters and reports are not edited. The original formatting of letters and referee reports may not be reflected in this compilation.)

DOI: https://doi.org/10.1128/spectrum.03183-25

Re: Spectrum03183-25 (**Age-Driven Shift of the Camel Gut Microbiome and Resistome in an Antibiotic-Free Environment**)

Dear Dr. Haihong Hao:

Thank you for the privilege of reviewing your work. Below you will find my comments, instructions from the Spectrum editorial office, and the reviewer comments.

Please return the manuscript within 60 days; if you cannot complete the modification within this time period, please contact me. If you do not wish to modify the manuscript and prefer to submit it to another journal, notify me immediately so that the manuscript may be formally withdrawn from consideration by Spectrum.

Revision Guidelines
To submit your modified manuscript, log into the submission site at https://spectrum.msubmit.net/cgi-bin/main.plex. Go to Author Tasks and click the appropriate manuscript title to begin. The information you entered when you first submitted the paper will be displayed; update this as necessary. Note the following requirements:

• Upload point-by-point responses to the issues raised by the reviewers in a file named "Response to Reviewers," NOT in your cover letter.
• Upload a compare copy of the manuscript (without figures) as a "Marked-Up Manuscript" file.
• Upload a clean .DOC/.DOCX version of the revised manuscript and remove the previous version.
• Each figure must be uploaded as a separate, editable, high-resolution file (TIFF or EPS preferred), and any multipanel figures must be assembled into one file.
• Any supplemental material intended for posting by ASM should be uploaded with their legends separate from the main manuscript. You can combine all supplemental material into one file (preferred) or split it into a maximum of 10 files with all associated legends included.

For complete guidelines on revision requirements, see our Submission and Review Process webpage. Submission of a paper that does not conform to guidelines may delay acceptance of your manuscript.

Data availability: ASM policy requires that data be available to the public upon online posting of the article, so please verify all links to sequence records, if present, and make sure that each number retrieves the full record of the data. If a new accession number is not linked or a link is broken, provide Spectrum production staff with the correct URL for the record. If the accession numbers for new data are not publicly accessible before the expected online posting of the article, publication may be delayed; please contact production staff (Spectrum@asmusa.org) immediately with the expected release date.

Publication Fees: For information on publication fees and which article types are subject to charges, visit our website. If your manuscript is accepted for publication and any fees apply, you will be contacted separately about payment during the production process; please follow the instructions in that e-mail. Arrangements for payment must be made before your article is published.

ASM Membership: Corresponding authors may join or renew ASM membership to obtain discounts on publication fees. Need to upgrade your membership level? Please contact Customer Service at Service@asmusa.org.

The ASM Journals program strives for constant improvement in our submission and publication process. Please tell us how we can improve your experience by taking this quick Author Survey.

Thank you for submitting your paper to Spectrum.

Sincerely,
Hao-Yu Liu
Editor
Microbiology Spectrum

Reviewer #1 (Comments for the Author):

This manuscript presents a metagenomic investigation into age-related changes in the gut microbiome, resistome, and mobilome of camels raised in an antibiotic-free context. The topic is timely and relevant within offering insights into the natural dynamics of antimicrobial resistance genes (ARGs) in a livestock species with minimal anthropogenic antibiotic exposure. The study design is appropriate, the methodological description is generally clear, and the integration of microbiome, resistome,

CAZyme, and mobilome data provides a multidimensional perspective. The findings that juveniles harbor a more diverse and mobile resistome than adults are interesting and contribute to understanding how host development shapes microbial communities and resistance reservoirs independently of drug selection pressure. The manuscript contains valuable data and addresses an important research gap, but requires significant revisions to strengthen the evidence for its central claim.

1.The manuscript's title and central premise rely on the environment being antibiotic-free. The Methods state only that "None of the animals had received antibiotics before or during sample collection." This does not constitute proof of an "antibiotic-free environment." Action Required: The authors must either: a) Provide Evidence: Include data to robustly support the "antibiotic-free" claim, or b) Moderate the Language: Change the title and text throughout to more accurately reflect the reality.

2.The abstract states adults had a "narrower resistome," but the Discussion (Paragraph 2) argues adult resistomes have "higher functional diversity." This contradiction must be resolved.

3.The claim that overall microbiome and ARG diversity were "moderately correlated (Spearman's r = 0.42)" appears unsupported. The relevant results (Fig. 2E-G) show negative correlations between ARG metrics (Shannon, Richness, Pielou) and microbiome diversity metrics. The text and figures need careful reconciliation.

4.Some references seem tangential or incorrectly applied (e.g., Ref 1 on swine antibiotics; Ref 20 on West Nile virus isolation from camels). References should be directly relevant to the point being made.

5.The discussion frequently cites studies in infants, goats, and cattle. While useful, a more focused discussion on the limited existing literature about camel gut microbiomes and resistomes would better contextualize the novelty of this work.

6.The "Results" section is long and mixes high-level observations with detailed statistical outcomes, making it difficult to follow. Consider streamlining and focusing on the main trends.

7.The manuscript requires thorough language editing for fluency and precision. There are instances of awkward phrasing, run-on sentences, and minor grammatical errors.

8.The reference list format is inconsistent. All references must conform strictly to the target journal's style guide.

9.The distinction between transposases and insertion sequences (IS elements) is central to the conclusion of age-related mobilome shifts. However, the manuscript does not explicitly describe the criteria used to differentiate these MGE types during annotation.

10.Some figures (e.g., Fig. 4B, 5C) lack detailed legends explaining node size, color coding, and statistical thresholds. Enhance figure legends to include explicit descriptions of these elements to facilitate independent interpretation.

11.The Discussion effectively links results to existing literature but could be streamlined to emphasize novel findings.

12.It does not mention whether the non-redundant gene catalog, ARG annotations, or MGE datasets are publicly available. Please specify if these derived datasets are accessible and provide additional accession numbers or repository links if applicable.

Reviewer #2 (Comments for the Author):

This study addresses a critical gap in understanding age-driven microbiome/resistome dynamics in an antibiotic-free model (camels), with a generally rigorous experimental design and comprehensive methodology-though further revisions and improvements are needed.

Suggestions for Improvement:

(1) Only two age cohorts miss sub-adult stages. Suggestion: If it is possible should add a sub-adult group or use a longitudinal design to track individual camels across development.

(2) Diet is a key driver of gut microbiome shifts, but the study provides limited detail on whether juvenile and adult diets were standardized.

(3) Fecal samples contain gastrointestinal transient bacteria and contaminating bacteria, and the sequencing results cannot truly reflect the intestinal colonizing bacteria. It is recommended to clearly explain how these transient bacteria and contaminating bacteria are excluded.

This study provides valuable insights into age-dependent microbiome and resistome dynamics in a natural system, with a well-structured analytical pipeline. Addressing the above limitations-particularly filling age gaps will significantly enhance the rigor and generalizability of the findings. The work's focus on antibiotic-free hosts also aligns with One Health priorities, making these improvements highly impactful.

This manuscript presents a metagenomic investigation into age-related changes in the gut microbiome, resistome, and mobilome of camels raised in an antibiotic-free context. The topic is timely and relevant within offering insights into the natural dynamics of antimicrobial resistance genes (ARGs) in a livestock species with minimal anthropogenic antibiotic exposure. The study design is appropriate, the methodological description is generally clear, and the integration of microbiome, resistome, CAZyme, and mobilome data provides a multidimensional perspective. The findings that juveniles harbor a more diverse and mobile resistome than adults are interesting and contribute to understanding how host development shapes microbial communities and resistance reservoirs independently of drug selection pressure. The manuscript contains valuable data and addresses an important research gap, but requires significant revisions to strengthen the evidence for its central claim.

1. The manuscript's title and central premise rely on the environment being antibiotic-free. The Methods state only that "None of the animals had received antibiotics before or during sample collection." This does not constitute proof of an "antibiotic-free environment." Action Required: The authors must either: a) Provide Evidence: Include data to robustly support the "antibiotic-free" claim, or b) Moderate the Language: Change the title and text throughout to more accurately reflect the reality.
2. The abstract states adults had a "narrower resistome," but the Discussion (Paragraph 2) argues adult resistomes have "higher functional diversity." This contradiction must be resolved.
3. The claim that overall microbiome and ARG diversity were "moderately correlated (Spearman's r = 0.42)" appears unsupported. The relevant results (Fig. 2E-G) show negative correlations between ARG metrics (Shannon, Richness, Pielou) and microbiome diversity metrics. The text and figures need careful reconciliation.
4. Some references seem tangential or incorrectly applied (e.g., Ref 1 on swine antibiotics; Ref 20 on West Nile virus isolation from camels). References should be directly relevant to the point being made.
5. The discussion frequently cites studies in infants, goats, and cattle. While useful, a more focused discussion on the limited existing literature about camel gut microbiomes and resistomes would better contextualize the novelty of this work.
6. The "Results" section is long and mixes high-level observations with detailed statistical outcomes, making it difficult to follow. Consider streamlining and focusing on the main trends.
7. The manuscript requires thorough language editing for fluency and precision. There are instances of awkward phrasing, run-on sentences, and minor grammatical errors.
8. The reference list format is inconsistent. All references must conform strictly to the target journal's style guide.
9. The distinction between transposases and insertion sequences (IS elements) is central to the conclusion of age-related mobilome shifts. However, the manuscript does not explicitly describe the criteria used to differentiate these MGE types during annotation.
10. Some figures (e.g., Fig. 4B, 5C) lack detailed legends explaining node size, color coding, and statistical thresholds. Enhance figure legends to include explicit descriptions of these elements to facilitate independent interpretation.
11. The Discussion effectively links results to existing literature but could be streamlined to

emphasize novel findings.

12. It does not mention whether the non-redundant gene catalog, ARG annotations, or MGE datasets are publicly available. Please specify if these derived datasets are accessible and provide additional accession numbers or repository links if applicable.

This study addresses a meaningful question with a suitable multi-omics approach and generates data of potential interest to the fields of microbial ecology, antimicrobial resistance, and livestock microbiome research. However, the manuscript in its current form is not acceptable for publication due to the critical, unsubstantiated "antibiotic-free" claim and significant inconsistencies between the text, figures, and data interpretation.

Decision: reject

Note to the Editor: If the authors can convincingly address the central issue regarding the "antibiotic-free" premise and rigorously correct the numerous textual and graphical inconsistencies, this manuscript has the potential to be a valuable contribution. A re-review would be necessary.

Comments:

This study addresses a critical gap in understanding age-driven microbiome/resistome dynamics in an antibiotic-free model (camels), with a generally rigorous experimental design and comprehensive methodology—though further revisions and improvements are needed.

Suggestions for Improvement:

(1) Only two age cohorts miss sub-adult stages. Suggestion: If it is possible should add a sub-adult group or use a longitudinal design to track individual camels across development.

(2) Diet is a key driver of gut microbiome shifts, but the study provides limited detail on whether juvenile and adult diets were standardized.

(3) Fecal samples contain gastrointestinal transient bacteria and contaminating bacteria, and the sequencing results cannot truly reflect the intestinal colonizing bacteria. It is recommended to clearly explain how these transient bacteria and contaminating bacteria are excluded.

This study provides valuable insights into age-dependent microbiome and resistome dynamics in a natural system, with a well-structured analytical pipeline. Addressing the above limitations—particularly filling age gaps will significantly enhance the rigor and generalizability of the findings. The work's focus on antibiotic-free hosts also aligns with One Health priorities, making these improvements highly impactful.

Response to Editor and Reviewers

Manuscript ID: Spectrum03183-25

We sincerely thank the Editor and both Reviewers for their careful evaluation of our manuscript and for the constructive comments that helped us to improve the clarity and strength of our work. We have revised the manuscript accordingly. Below we provide a point-by-point response, with all changes marked in the revised manuscript.

Reviewer #1

1.The manuscript's title and central premise rely on the environment being antibiotic-free. The Methods state only that "None of the animals had received antibiotics before or during sample collection." This does not constitute proof of an "antibiotic-free environment." Action Required: The authors must either: a) Provide Evidence: Include data to robustly support the "antibiotic-free" claim, or b) Moderate the Language: Change the title and text throughout to more accurately reflect the reality.

Response:

We appreciate this important comment and fully agree that our original wording overstated the evidence for an “antibiotic-free environment”. We have moderated the language throughout the manuscript.

First, we removed the phrase “in an antibiotic-free environment” from the title, which now reads: “Age-Driven Shifts of the Camel Gut Microbiome and Resistome in Extensively Reared Dromedary Camels”. Second, in the Abstract, Introduction, Discussion, and Conclusion, we replaced “antibiotic-free environment/context” and “absence of antibiotic pressure” with more cautious terms such as “extensive grazing systems with no recorded antibiotic treatments”, “minimal documented anthropogenic antibiotic pressure” and “apparent absence of direct antibiotic selection pressure”(lines85-89).

In the Methods (Sample collection), we also added details on how information on antibiotic use was obtained. Specifically, we now state that there were no recorded

therapeutic or prophylactic antibiotic treatments for the enrolled camels according to farm records and interviews with local veterinarians and animal owners, and that routine in-feed medication is not practiced in these extensive grazing systems. At the same time, we explicitly acknowledge that we did not measure antibiotic residues in feed, water or the environment and that unrecorded or environmental exposure cannot be excluded (lines92-104).

We believe these revisions more accurately represent our data and address the reviewer’s concern about the strength of our original “antibiotic-free” claim.

2.The abstract states adults had a "narrower resistome," but the Discussion (Paragraph 2) argues adult resistomes have "higher functional diversity." This contradiction must be resolved.

Response:

We thank the reviewer for highlighting this inconsistency between the Abstract and Discussion. Our quantitative analyses (Fig. 3A and Table S1) show that juvenile camels have significantly higher ARG richness and Shannon diversity than adults, indicating a broader and more diverse resistome in early life. The phrase “higher functional diversity” for adults in the Discussion was therefore incorrect and misleading.

We have now revised the text to ensure conceptual and terminological consistency throughout the manuscript. In the Abstract, we clarify that adults harbor a narrower and more functionally focused resistome dominated by a limited set of resistance classes. In the Discussion (Paragraph 2), we removed the incorrect statement that adults have higher functional diversity and instead emphasize that adult resistomes exhibit reduced ARG richness and functional diversity relative to juveniles, but are more specialized and compositionally stable(lines441-445).

We believe these changes resolve the contradiction and more accurately reflect the patterns shown in Fig. 3.

3.The claim that overall microbiome and ARG diversity were "moderately correlated (Spearman's r = 0.42)" appears unsupported. The relevant results (Fig. 2E-G) show negative correlations between ARG metrics (Shannon,

Richness, Pielou) and microbiome diversity metrics. The text and figures need careful reconciliation.

Response:

We thank the reviewer for pointing out this inconsistency. The statement in the Discussion that “overall microbiome and ARG diversities were moderately correlated (Spearman’s r = 0.42)” was an error left from an earlier draft and does not reflect the final correlation analyses. As correctly shown in Fig. 2E–G, all correlations between microbiome and ARG diversity metrics are negative (Shannon: r = –0.43, p = 0.0134; richness: r = –0.59, p = 0.0003; Pielou’s evenness: r = –0.41, p = 0.0168).

We have now revised the text to reconcile the Results and Discussion. In the Results section, we describe these as significant negative associations with the correct Spearman’s r and p values (Fig. 2E–G). In the Discussion, the incorrect sentence has been replaced with a description stating that overall microbiome and ARG diversity metrics show moderate negative correlations, with Spearman’s r ranging from –0.41 to –0.59, and we emphasize that samples with high ARG richness can exhibit relatively low taxonomic diversity, consistent with these negative associations(lines480-483).

4.Some references seem tangential or incorrectly applied (e.g., Ref 1 on swine antibiotics; Ref 20 on West Nile virus isolation from camels). References should be directly relevant to the point being made.

Response:

We thank the reviewer for this helpful observation. We agree that in the original version some references were not optimally matched to the statements they were intended to support. We have therefore carefully re-examined all citations in the manuscript and revised them to ensure that each reference is directly relevant to the corresponding text.

Specifically, the previous Ref 1, which focused on antibiotic use in swine, was originally cited in the general statement that “antimicrobial resistance is a growing global health threat to both human and animal populations.” In the revised manuscript this has been replaced by a more appropriate, broad overview of the global AMR

burden (lines 623-625).

Similarly, the previous Ref 20, which reported West Nile virus isolation from camels, was incorrectly cited to support a sentence describing the pseudo-ruminant forestomach and fermentation-based energy extraction in camels. We have removed this inappropriate citation and now cite studies that specifically address camel digestive physiology and foregut fermentation(lines 693-697).

5.The discussion frequently cites studies in infants, goats, and cattle. While useful, a more focused discussion on the limited existing literature about camel gut microbiomes and resistomes would better contextualize the novelty of this work.

Response:

We thank the reviewer for this insightful suggestion. We agree that our original Discussion relied too heavily on studies in infants and other ruminants and did not sufficiently anchor our findings in the context of the limited literature on camel gut microbiomes.

In the revised manuscript, we have substantially strengthened the camel-specific context in both the Introduction and Discussion. Specifically, we added a dedicated paragraph in the Introduction summarizing current knowledge on camel gut microbiota (taxonomic composition, fermentation capacity, and the very limited information available on ARGs and MGEs) and highlighting the major knowledge gap regarding age-related dynamics of the camel resistome (lines76-78) .

In the Discussion, we now include a focused paragraph that compares our results with the few existing studies on dromedary and Bactrian camel gut/foregut microbiomes, emphasizing consistencies (lines441-469) .

6. The "Results" section is long and mixes high-level observations with detailed statistical outcomes, making it difficult to follow. Consider streamlining and focusing on the main trends.

Response:

We have streamlined the Results to improve readability.

The Results section has been reorganized into clearer subsections (e.g.,

microbiome composition, CAZymes, resistome dynamics, ARG clusters, MGE patterns, and ARG co-selection). Redundant detailed statistics that were already presented in figure legends or Supplementary Tables have been shortened or removed from the main text.

7.The manuscript requires thorough language editing for fluency and precision. There are instances of awkward phrasing, run-on sentences, and minor grammatical errors.

Response:

Run-on sentences and awkward phrasing were corrected, and overly long paragraphs were split where appropriate.We hope the revised version reads much more fluently.

8.The reference list format is inconsistent. All references must conform strictly to the target journal's style guide.

Response:

Thank you for pointing out the inconsistency in the reference list format. We have carefully reformatted the entire reference list in strict accordance with the style guidelines of Microbiology Spectrum. Specifically, all key elements of the references, including author names, publication years, article titles, journal names, volumes, pages, and DOIs, are now presented in a fully consistent format across all entries

9.The distinction between transposases and insertion sequences (IS elements) is central to the conclusion of age-related mobilome shifts. However, the manuscript does not explicitly describe the criteria used to differentiate these MGE types during annotation.

Response:

We thank the reviewer for pointing out this important omission. In the original version, the criteria used to distinguish transposases from insertion sequences (IS elements) during MGE annotation were not explicitly described.

In the revised manuscript, we have now added a dedicated description of our MGE annotation and classification procedure in the Materials and methods section. Briefly, predicted protein-coding genes in the non-redundant catalog were searched

against MobileOG-db using DIAMOND blastp, and each significant hit was assigned to the MGE type reported in the MobileOG-db annotation. For the purpose of this study, we then collapsed MobileOG-db categories into broader classes: (i) "transposases", comprising genes annotated as transposase or transposition-related proteins that did not carry an insertion sequence (IS) family designation; (ii) "insertion sequences (IS elements)", comprising genes annotated as IS element or IS-family transposase, or bearing canonical IS family names in the MobileOG-db description; and (iii) integrase/relaxase and other conjugative transfer proteins, grouped as integrative/conjugative elements.

10.Some figures (e.g., Fig. 4B, 5C) lack detailed legends explaining node size, color coding, and statistical thresholds. Enhance figure legends to include explicit descriptions of these elements to facilitate independent interpretation.

Response:

We thank the reviewer for this helpful comment. We agree that the original legends for Fig. 4B and Fig. 5C did not fully explain all visual elements. In the revised manuscript, we have expanded these legends to explicitly describe what the node/segment size and color represent and to state the statistical thresholds used for constructing the networks.

For Fig. 4B, we now clarify that the Sankey diagram depicts the taxonomic lineage of all ARG-annotated genes from kingdom to species, that the width of each ribbon and the height of each segment are proportional to the number of ARG-annotated genes in that taxon, and that colors indicate different bacterial phyla. For Fig. 5C, we specify that nodes represent ARGs and different MGE types, that node colors differentiate ARGs from integrases, transposases, IS elements and other MGEs, and that edges represent significant positive co-occurrence correlations above the defined SparCC correlation and P-value thresholds.

11. The Discussion effectively links results to existing literature but could be streamlined to emphasize novel findings.

Response:

Thank you for your valuable suggestion to streamline the Discussion and

emphasize novel findings. We have shortened and refocused this section as recommended:

The first two paragraphs now succinctly highlight the main novel contributions of our study: age-related contraction and focusing of the resistome, shifts in dominant resistance mechanisms, and distinct mobilome strategies between juvenile and adult camels. Redundant explanations have been condensed, and camel-specific insights are now emphasized upfront before presenting cross-species comparisons, ensuring our unique findings take center stage.

12. It does not mention whether the non-redundant gene catalog, ARG annotations, or MGE datasets are publicly available. Please specify if these derived datasets are accessible and provide additional accession numbers or repository links if applicable.

Response:

We thank the reviewer for raising this point. In the original version, we only indicated the deposition of the raw metagenomic reads and did not explicitly describe the status of the derived datasets (non-redundant gene catalog, ARG and MGE annotations).

In our study, all downstream analyses are based on the raw metagenomic reads, which have been deposited in the NCBI Sequence Read Archive under BioProject accession PRJNA1256226, together with the software tools, reference databases and parameter settings described in the Materials and methods. The non-redundant camel gut gene catalog and the corresponding ARG and MGE annotation tables were generated as intermediate outputs by the sequencing provider's in-house pipeline and are not archived separately in a public repository.

Reviewer #2 :

(1) Only two age cohorts miss sub-adult stages. Suggestion: If it is possible should add a sub-adult group or use a longitudinal design to track individual camels across development.

Response:

We fully agree that including sub-adult camels and/or adopting a longitudinal design would provide a more complete picture of developmental dynamics. However, this was not feasible within the current project, which was conducted under field conditions in an extensive grazing system. Access to intermediate age classes was limited by farm management and animal movement, so we focused on obtaining sufficiently powered juvenile and adult cohorts. We now explicitly acknowledge this as a limitation in the Discussion and clarify that our conclusions pertain to cross-sectional differences between early- and late-stage animals. We also highlight the inclusion of sub-adult groups and longitudinal follow-up as important directions for future research.

(2) **Diet is a key driver of gut microbiome shifts, but the study provides limited detail on whether juvenile and adult diets were standardized.**

Response:

We agree with the reviewer that diet is a major determinant of gut microbiome composition. The animals in our study were kept under field conditions in an extensive grazing system, so the diet could not be experimentally standardized in a strict sense. However, all juvenile (SM) and adult (SY) camels belonged to the same herd, were managed together on a pasture-based farm in Xinjiang, and grazed on the same natural desert steppe vegetation dominated by camel thorn and desert bunchgrasses. During the winter season, both age groups also received the same supplemental alfalfa hay to support maintenance under harsh climatic conditions. No commercial concentrates or medicated feeds containing antibiotics were provided.

Thus, the main dietary difference between age groups reflects the normal age-appropriate feeding pattern in this production system: juvenile camels had free access to maternal milk while gradually transitioning to the same pasture and alfalfa hay as their dams, whereas adults relied exclusively on the forage-based diet (pasture plus alfalfa hay). To address the reviewer’s concern, we have added a more detailed description of herd management and feeding conditions in the Materials and methods (Animals, management and diet) section, and in the Discussion we explicitly acknowledge that age and diet are partially intertwined in this real-world setting. We

note that the age-associated patterns we describe should therefore be interpreted as reflecting combined effects of host development and the associated transition from milk-supported to fully forage-based diets under shared grazing and supplementation conditions.

(3) Fecal samples contain gastrointestinal transient bacteria and contaminating bacteria, and the sequencing results cannot truly reflect the intestinal colonizing bacteria. It is recommended to clearly explain how these transient bacteria and contaminating bacteria are excluded.

Response:

We thank the reviewer for this important comment. We agree that fecal metagenomes represent a mixture of resident gut colonizers, transient bacteria derived from feed and the environment, and potential low-level contaminants, and that this needs to be clearly addressed.

In the present study, our primary aim was to characterize age-associated patterns in the overall gut microbial, resistome and mobilome profiles of camels under real-world grazing conditions, rather than to define an exhaustive set of permanently colonizing taxa. Nevertheless, we took several steps to minimize external contamination and to focus our analyses on reproducible community members. First, fresh fecal pellets were collected in the field immediately after defecation, and pellets with visible contact with soil or standing water were avoided. Using sterile gloves and forceps, we subsampled the inner portion of each pellet into sterile tubes, which were then rapidly cooled and stored at −80 °C until DNA extraction. All DNA extraction and library preparation steps were performed with sterile consumables and standard clean bench procedures to reduce laboratory contamination. Second, in the bioinformatic pipeline, raw reads were subjected to quality filtering and host-read removal before taxonomic, ARG and MGE profiling, so that downstream analyses were based on high-quality microbial sequences.

We have now added a more detailed description of these sampling and processing procedures in the Materials and methods (Animals, sampling and DNA extraction; Metagenomic processing and taxonomic profiling) sections. In addition,

we explicitly acknowledge in the Discussion that fecal metagenomes capture both resident and transient lumen-associated bacteria and that it is not possible to completely separate these components from sequence data alone. Accordingly, our age-related results should be interpreted as patterns in the fecal gut ecosystem under shared management conditions rather than as a strict catalogue of permanent colonizers.

Re: Spectrum03183-25R1 (**Age-Driven Shifts of the Camel Gut Microbiome and Resistome in Extensively Reared Dromedary Camels**)

Dear Dr. chencheng xiao:

Thank you for the privilege of reviewing your work. Below you will find my comments, instructions from the Spectrum editorial office, and the reviewer comments.

Please return the manuscript within 60 days; if you cannot complete the modification within this time period, please contact me. If you do not wish to modify the manuscript and prefer to submit it to another journal, notify me immediately so that the manuscript may be formally withdrawn from consideration by Spectrum.

Revision Guidelines
To submit your modified manuscript, log into the submission site at https://spectrum.msubmit.net/cgi-bin/main.plex. Go to Author Tasks and click the appropriate manuscript title to begin. The information you entered when you first submitted the paper will be displayed; update this as necessary. Note the following requirements:

• Upload point-by-point responses to the issues raised by the reviewers in a file named "Response to Reviewers," NOT in your cover letter.
• Upload a compare copy of the manuscript (without figures) as a "Marked-Up Manuscript" file.
• Upload a clean .DOC/.DOCX version of the revised manuscript and remove the previous version.
• Each figure must be uploaded as a separate, editable, high-resolution file (TIFF or EPS preferred), and any multipanel figures must be assembled into one file.
• Any supplemental material intended for posting by ASM should be uploaded with their legends separate from the main manuscript. You can combine all supplemental material into one file (preferred) or split it into a maximum of 10 files with all associated legends included.

For complete guidelines on revision requirements, see our Submission and Review Process webpage. Submission of a paper that does not conform to guidelines may delay acceptance of your manuscript.

Data availability: ASM policy requires that data be available to the public upon online posting of the article, so please verify all links to sequence records, if present, and make sure that each number retrieves the full record of the data. If a new accession number is not linked or a link is broken, provide Spectrum production staff with the correct URL for the record. If the accession numbers for new data are not publicly accessible before the expected online posting of the article, publication may be delayed; please contact production staff (Spectrum@asmusa.org) immediately with the expected release date.

Publication Fees: For information on publication fees and which article types are subject to charges, visit our website. If your manuscript is accepted for publication and any fees apply, you will be contacted separately about payment during the production process; please follow the instructions in that e-mail. Arrangements for payment must be made before your article is published.

ASM Membership: Corresponding authors may join or renew ASM membership to obtain discounts on publication fees. Need to upgrade your membership level? Please contact Customer Service at Service@asmusa.org.

The ASM Journals program strives for constant improvement in our submission and publication process. Please tell us how we can improve your experience by taking this quick Author Survey.

Thank you for submitting your paper to Spectrum.

Sincerely,
Hao-Yu Liu
Editor
Microbiology Spectrum

Reviewer #1 (Comments for the Author):

This is a well-designed study with clear scientific value. The article systematically reveals the synergistic changes in the intestinal microbiome, resistance group and mobile genetic elements of camels with the age of the host under grazing conditions with low antibiotic exposure. The research fills the gap in the study of the age-stratified intestinal microecology and drug resistance gene pool of the camel, a characteristic livestock species, providing valuable data and insights for understanding how

host development naturally shapes the intestinal flora and drug resistance gene ecology. The main structure of the article is complete, the logic is clear, and the data presentation is relatively rich.The manuscript contains valuable data and addresses an important research gap, but requires revisions to strengthen the evidence for its central claim.

1. The abstract lacks precision: Inconsistent descriptions regarding the extent of "antibiotic use" (e.g., "limited recorded antibiotic use" vs. "low documented antibiotic exposure") are present, with the latter potentially carrying a broader meaning. Terminology should be unified and strictly aligned with the core finding ("no recorded therapeutic antibiotic treatments").
2. The definition of age is ambiguous: The definition "juvenile ({less than or equal to} 2 years)" in the abstract is inconsistent with the specific sample age (6 months) mentioned in the results section. This requires clarification and standardization.
3. Inconsistent professional terminology: The entire text should uniformly adopt currently accepted nomenclature in microbial taxonomy (e.g., Bacillota, Pseudomonadota). When older names (e.g., Firmicutes, Proteobacteria) first appear, they should be indicated in parentheses, with consistency maintained thereafter.
4. While emphasizing "no recorded therapeutic antibiotic use" and implementing measures such as "avoiding contact with soil/standing water," the study does not specify whether antibiotic residues in feed, drinking water, or the environment were tested. It also fails to clearly state whether field blank controls were set up to monitor potential cross-contamination.
5. The study found enrichment of vanW/vanY genes in foals but did not discuss or test whether feed contained common additives (e.g., virginiamycin, monensin) that might potentially cross-select for such genes, leaving the interpretation of the conclusions incomplete.

This is a well-designed study with clear scientific value. The article systematically reveals the synergistic changes in the intestinal microbiome, resistance group and mobile genetic elements of camels with the age of the host under grazing conditions with low antibiotic exposure. The research fills the gap in the study of the age-stratified intestinal microecology and drug resistance gene pool of the camel, a characteristic livestock species, providing valuable data and insights for understanding how host development naturally shapes the intestinal flora and drug resistance gene ecology. The main structure of the article is complete, the logic is clear, and the data presentation is relatively rich.The manuscript contains valuable data and addresses an important research gap, but requires revisions to strengthen the evidence for its central claim.

- The abstract lacks precision: Inconsistent descriptions regarding the extent of "antibiotic use" (e.g., "limited recorded antibiotic use" vs. "low documented antibiotic exposure") are present, with the latter potentially carrying a broader meaning. Terminology should be unified and strictly aligned with the core finding ("no recorded therapeutic antibiotic treatments").
- The definition of age is ambiguous: The definition "juvenile (≤ 2 years)" in the abstract is inconsistent with the specific sample age (6 months) mentioned in the results section. This requires clarification and standardization.
- Inconsistent professional terminology: The entire text should uniformly adopt currently accepted nomenclature in microbial taxonomy (e.g., Bacillota, Pseudomonadota). When older names (e.g., Firmicutes, Proteobacteria) first appear, they should be indicated in parentheses, with consistency maintained thereafter.
- While emphasizing "no recorded therapeutic antibiotic use" and implementing measures such as "avoiding contact with soil/standing water," the study does not specify whether antibiotic residues in feed, drinking water, or the environment were tested. It also fails to clearly state whether field blank controls were set up to monitor potential cross-contamination.
- The study found enrichment of vanW/vanY genes in foals but did not discuss or test whether feed contained common additives (e.g., virginiamycin, monensin) that might potentially cross-select for such genes, leaving the interpretation of the conclusions incomplete.

Response to Reviewer 1

We sincerely thank the reviewer for the careful and constructive evaluation of our manuscript. We have revised the manuscript in response to the reviewer’s comments to improve clarity, consistency, and interpretation.

Specifically, we have unified terminology related to antibiotic exposure, clarified age definitions, updated microbial taxonomic nomenclature, and expanded the Methods and Discussion to better address methodological scope and interpretation. These revisions are detailed below.

Comment 1:The abstract lacks precision: Inconsistent descriptions regarding the extent of "antibiotic use" (e.g., "limited recorded antibiotic use" vs. "low documented antibiotic exposure") are present, with the latter potentially carrying a broader meaning. Terminology should be unified and strictly aligned with the core finding ("no recorded therapeutic antibiotic treatments").

Response: We thank the reviewer for pointing out this inconsistency. We agree that the terminology used to describe antibiotic exposure should be precise and consistent throughout the manuscript. We have therefore revised the Abstract and related sections to uniformly use the phrase “no recorded therapeutic antibiotic treatments”, which accurately reflects farm records and veterinary reports for the enrolled animals. All broader or potentially ambiguous expressions (e.g., “limited recorded antibiotic

use" and "low documented antibiotic exposure") have been replaced accordingly to ensure clarity and consistency.

Comment 2: The definition of age is ambiguous: The definition "juvenile ({less than or equal to} 2 years)" in the abstract is inconsistent with the specific sample age (6 months) mentioned in the results section. This requires clarification and standardization.

Response: We thank the reviewer for this helpful comment. We agree that the age definition for juvenile camels should be stated consistently and more precisely. We have therefore revised the Abstract to explicitly reflect the actual sampled age of juvenile animals (approximately 6 months), and clarified in the main text that the term "juvenile" in this study refers to camels sampled at this early developmental stage. These revisions ensure consistency between the Abstract, Results, and Materials and Methods sections. These changes were made in the Abstract (age definition) and clarified in the Materials and Methods section to ensure consistency across the manuscript.

Comment 3: Inconsistent professional terminology: The entire text should uniformly adopt currently accepted nomenclature in microbial taxonomy (e.g., Bacillota, Pseudomonadota). When older names (e.g., Firmicutes, Proteobacteria) first appear, they should be indicated in parentheses, with consistency maintained thereafter.

Response: We thank the reviewer for this helpful suggestion. We have

revised the manuscript to consistently adopt the currently accepted microbial taxonomic nomenclature throughout the text. On first mention, updated phylum names are now provided together with their legacy terms in parentheses (e.g., Bacillota (formerly Firmicutes), Pseudomonadota (formerly Proteobacteria)). Subsequent references use only the updated nomenclature. Similar revisions were applied to other major bacterial phyla to ensure consistency across the manuscript.

Manuscript changes: Updated taxonomic names were implemented throughout the Results, Discussion, and figure legends to ensure uniform and current nomenclature.

Comment 4: While emphasizing "no recorded therapeutic antibiotic use" and implementing measures such as "avoiding contact with soil/standing water," the study does not specify whether antibiotic residues in feed, drinking water, or the environment were tested. It also fails to clearly state whether field blank controls were set up to monitor potential cross-contamination.

We thank the reviewer for this important comment. Antibiotic residues in feed, drinking water, or the surrounding environment were not directly measured in this study. According to farm management records and veterinary reports, no therapeutic or prophylactic antibiotics were administered to the enrolled camels, and no medicated feeds were used during the study period.

During sampling, procedures were applied to minimize environmental contamination, and a negative extraction control was included during DNA extraction. We acknowledge that field blank controls were not included and that low-level or unrecorded environmental exposure to antibiotics cannot be completely excluded. These points are now explicitly stated in the Materials and Methods and discussed as a limitation.

Comment 5: The study found enrichment of vanW/vanY genes in foals but did not discuss or test whether feed contained common additives (e.g., virginiamycin, monensin) that might potentially cross-select for such genes, leaving the interpretation of the conclusions incomplete.

Response: We agree that potential co-selection by feed additives is a plausible consideration; however, in our dataset the vanW/vanY enrichment occurs alongside a juvenile resistome architecture characterized by stronger associations with transposase-dominated mobile genetic elements. We therefore interpret this pattern as consistent with enhanced early-life horizontal gene transfer and genome plasticity during microbiome assembly, rather than attributing it to a specific selective agent. At the same time, we acknowledge that specific external drivers, including feed additives, were not directly measured and therefore cannot be completely excluded.

Re: Spectrum03183-25R2 (**Age-Driven Shifts of the Camel Gut Microbiome and Resistome in Extensively Reared Dromedary Camels**)

Dear Dr. chencheng xiao:

Your manuscript has been accepted, and I am forwarding it to the ASM production staff for publication. Your paper will first be checked to make sure all elements meet the technical requirements. ASM staff will contact you if anything needs to be revised before copyediting and production can begin. Otherwise, you will be notified when your proofs are ready to be viewed.

Data Availability: ASM policy requires that data be available to the public upon online posting of the article, so please verify all links to sequence records, if present, and make sure that each number retrieves the full record of the data. If a new accession number is not linked or a link is broken, provide production staff with the correct URL for the record. If the accession numbers for new data are not publicly accessible before the expected online posting of the article, publication may be delayed; please contact ASM production staff immediately with the expected release date.

Publication Fees: For information on publication fees and which article types have charges, please visit our website. We have partnered with Copyright Clearance Center (CCC) to collect author charges. If fees apply to your paper, you will receive a message from no-reply@copyright.com with further instructions. For questions related to paying charges through RightsLink, please contact CCC at ASM_Support@copyright.com or toll free at +1-877-622-5543. CCC makes every attempt to respond to all emails within 24 hours.

ASM Membership: Corresponding authors may join or renew ASM membership to obtain discounts on publication fees. Need to upgrade your membership level? Please contact Customer Service at Service@asmusa.org.

Embargo Policy: A press release may be issued as soon as the manuscript is posted on the Spectrum Latest Articles webpage. The corresponding author will receive an email with the subject line "ASM Journals Author Services Notification" when the article is available online.

The ASM Journals program strives for constant improvement in our submission and publication process. Please tell us how we can improve your experience by taking this quick Author Survey.

Thank you for submitting your paper to Spectrum.

Sincerely,
Hao-Yu Liu
Editor
Microbiology Spectrum
